# Relcovaptan: a promising therapeutic agent in traumatic spinal cord injury that acts by modulating newly identified transcriptional regulators of aquaporins compared to tolvaptan

**DOI:** 10.55730/1300-0144.6097

**Published:** 2025-09-22

**Authors:** Şeyma İŞ, Duygu CEMAN, Merih İŞ, Neşe KESER, Şaban TEKİN

**Affiliations:** 1 Department of Basic Medical Sciences, Division of Medical Biology, Hamidiye School of Medicine, University of Health Sciences, İstanbul, Turkiye; 2 Department of Molecular Biotechnology, Division of Bioinformatics, Faculty of Science, Turkish-German University, İstanbul, Turkiye; 3 Neurosurgery Clinic, Haydarpasa Numune Training and Research Hospital, İstanbul, Turkiye; 4 Neurosurgery Clinic, Fatih Sultan Mehmet Training and Research Hospital, İstanbul, Turkiye; 5 Department of Surgical Medical Sciences, Division of Brain and Nerve Surgery, Hamidiye School of Medicine, University of Health Sciences, İstanbul, Turkiye; 6 Experimental Medicine Research and Application Center, University of Health Sciences, İstanbul, Turkiye

**Keywords:** Aquaporins, edema, relcovaptan, RNA sequencing, tolvaptan, traumatic spinal cord injury

## Abstract

**Background/aim:**

Every year, hundreds of thousands of people worldwide suffer from traumatic spinal cord injury (tSCI), which causes irreversible damage, edema, and inflammation. Despite its devastating impact, no safe and effective medication is available. Edema formation is one of the earliest pathological events in tSCI, beginning within minutes after injury. Cytotoxic edema progresses to vasogenic edema, exacerbating irreversible damage. Our study aimed to investigate a therapeutic approach targeting cytotoxic edema in the acute phase of tSCI to improve treatment outcomes. Aquaporins (AQPs) are crucial in edema formation and tSCI pathogenesis. Vasopressin, also known as the antidiuretic hormone, modulates *Aqp* expression and translocation by initiating cell signaling via vasopressin 1a receptor (V1aR) and vasopressin 2 receptor (V2R). We investigated the effects of two V1aR and V2R antagonists (relcovaptan and tolvaptan, respectively) on edema formation and *Aqp* expression in tSCI.

**Materials and methods:**

Transcriptome analysis was performed on male Sprague Dawley rats that were traumatized to determine the effect of relcovaptan and tolvaptan after tSCI. Gene set enrichment analysis and bioinformatics approaches identified critical genes and signaling pathways associated with the drug treatment.

**Results:**

According to our results, tolvaptan was not suitable for the treatment of tSCI. On the other hand, relcovaptan had multiple positive effects. These include not only the positive effect on edema, which is achieved by suppression of *Aqp1*, *Aqp4*, and *Aqp11* expression, but also immunoregulation, neuroprotection, neuroregeneration, and osmoregulation via activator protein-1 and galanin.

**Conclusion:**

Our study suggests that clinical application of relcovaptan may be a promising treatment option for improving tSCI.

## 1. Introduction

Spinal cord injury (SCI) is a devastating condition that affects patients and their families. The impact is not only physiological; it is also psychological, social, and financial [1]. About 250,000 to 500,000 new cases of SCI occur worldwide each year, 10% of which are due to tumors or neurodegenerative diseases such as multiple sclerosis or cervical spondylotic myelopathy, and the remaining 90% due to trauma (i.e. from traffic accidents, falls, or stab/gunshot wounds) [2–4]. Despite the high annual incidence of traumatic SCI (tSCI), significant challenges persist today, arising from its complex pathophysiology and limited treatment options. Furthermore, delays in diagnosis and treatment of tSCI can have severe consequences, including neurological damage, chronic pain, or deformity [5–8]. Additionally, there is still limited knowledge regarding the cellular and molecular mechanisms of tSCI, which consists of 2 phases: the primary injury mechanism and the secondary injury mechanism.

The primary injury mechanism is initial mechanical damage such as dislocation and fracture, fissure, or cut in the spinal cord (SC) because of trauma. Because injury occurs unexpectedly, it cannot be prevented or altered in severity [9]. As a result of the initial damage, the integrity of the vascular and neural systems is locally impaired at the injury site, resulting in ischemia and motor, sensory, and autonomic dysfunction [10,11]. The initial mechanical insult tends to primarily damage the central gray matter, with relative sparing of the white matter, especially peripherally. The increased propensity for damage to gray matter could result from its softer consistency and greater vascularity [12]. The secondary injury mechanism starts in the first posttraumatic moments and lasts for weeks or even months. It is subdivided into 3 phases: acute, subacute, and chronic. The acute phase involves pathophysiological processes such as ischemia, ion and electrolyte homeostasis disturbance, cytotoxic and vasogenic edema, loss of autoregulation, excitotoxicity, oxidative stress, lipid peroxidation, and inflammation. Axonal demyelination and degeneration, apoptosis, remodeling of the extracellular matrix, and the formation of a glial wound occur in the subacute phase. As the glial wound matures, a cystic cavity forms in the chronic phase [8,9,13–15]. Thus, the secondary injury mechanism leads to the self-destruction of the SC and impedes neuroregeneration [9,16].

Many molecular and cellular events occur during the acute phase of injury, including edema formation, that can be targeted to protect neural tissue and promote functional recovery. Therefore, therapeutic agents administered shortly after tSCI have long been explored [17]. In the context of edema, aquaporins (AQPs)—small, integral membrane proteins that transport water transcellularly—are promising targets [18,19]. To date, 13 AQP family members have been identified; however, only AQP1, AQP4, and AQP9 have been identified to play a role in edema following SCI [18,20–22].

However, although many potential pharmacological treatments have long been studied for tSCI, there is still no medication available [23]. Methylprednisolone has been used to treat tSCI since the late 1950s. However, its effectiveness has always been debated due to the lack of reproducible positive effects and serious side effects, such as pneumonia and even death [24,25]. On these grounds, methylprednisolone is no longer recommended by the American Association of Neurological Surgeons and has been removed from the treatment protocol [8,24,26]. There is still no medication available for neuroprotection and improved functional outcomes after tSCI [8,23,27]. The delay in diagnosis and treatment of tSCI can be costly, resulting in neurological injury, chronic pain, or deformity [5–8]. Therefore, the neurosurgical community and neuroscientists must work diligently to advocate for the prevention and treatment of tSCI around the world, and there is an urgent need to identify therapeutic drugs.

In this study, we specifically focused on edema formation by AQP regulation, one of the earliest events in the pathogenesis of tSCI that begins within minutes after injury [28–30]. Although the precise mechanisms remain unclear, inhibiting edema can markedly improve patient prognosis [18]. Secondary injury triggers activation of astrocytes, fibroblasts, pericytes, and microglia, with astrocytes expressing *Aqp4* in their end feet processes and being key regulators of water transport in the central nervous system [17,18]. Edema arises from both dysregulated water homeostasis (cytotoxic edema) and disruption of the blood–spinal cord barrier (BSCB) (vasogenic edema), with astrocytic AQP4 channels mediating water transport [18,21,31].

We aimed to investigate the effects of relcovaptan and tolvaptan, which are vasopressin 1a receptor (V1aR) and vasopressin 2 receptor (V2R) antagonists [32,33], respectively, focusing on their effect on *Aqp* expression after tSCI using transcriptome analyses. Our therapeutic strategy targets the prevention of cytotoxic edema at the onset of secondary injury, thereby limiting the progression to vasogenic edema and subsequent irreversible BSCB damage. To the best of our knowledge, this is among the first transcriptome-based analysis addressing edema modulation in tSCI.

## 2. Materials and methods

**Figure 1:**
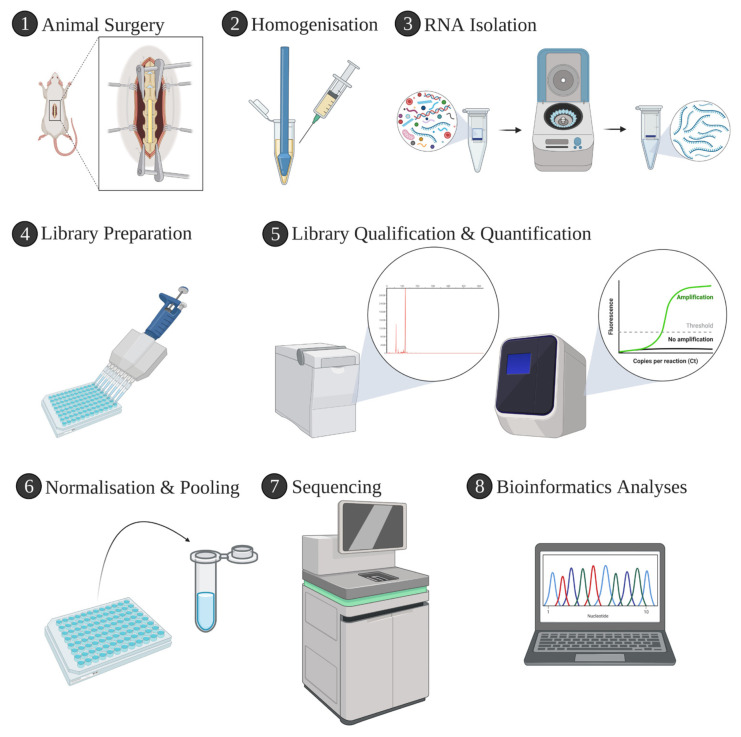
Overview of the study design. Created using BioRender.com.

The general overview of the study is shown in Figure 1.

### 2.1. Animals

We used 24 male Sprague Dawley rats (10–12 weeks old, mean weight of 250 g) in this study. These were divided into 3 groups with 8 animals each: the control group, the relcovaptan group, and the tolvaptan group. Animal care in and after surgical procedures was in accordance with the European Convention for the Protection of Vertebrate Animals used for Experimental and other Scientific Purposes (ETS number: 123).

### 2.2. Traumatic spinal cord injury

Rats were injected intraperitoneally (i.p.) with cefamezin (100 mg/kg) for surgical prophylaxis. Ketamine hydrochloride (100 mg/kg, i.p.) and xylazine hydrochloride (10 mg/kg, i.p.) were used to anesthetize the rats. After T9-T11 laminectomy, the SC was traumatized by clamping with a Yasargil aneurysm clip. The rats were injected i.p. with 2 mg/kg relcovaptan (SR 49059, CAS: 150375-75-0, Sigma-Aldrich, St. Louis, MO, USA) or 30 mg/kg tolvaptan (CAS: 150683-30-0, Sigma-Aldrich) 10 minutes post-trauma. Paracetamol (100 mg/kg, i.p.) was used every 8 h to relieve pain. Rats in the control group did not undergo surgical procedures; instead, they were only treated with one dose of 0.9% saline (2 mL/kg). Relcovaptan and tolvaptan have only previously been used for conditions such as traumatic brain injury and stroke. This means that they were used for SCI first time in our study. The doses of the agents were determined based on previous studies [34–36]. The animals were placed in a humidity- and temperature-controlled chamber. Rats were sacrificed by injecting sodium pentothal (100 mg/kg, i.p.) 24 h after injury, and SC segments containing the injury epicenter were harvested.

### 2.3. Neurological examination

The results of the neurological examination were assessed according to the motor function score of Gale et al. [37]. All behavioral tests were conducted by 2 blinded investigators. The order in which animals were tested for a particular task was determined randomly for each animal. The motor function scores were assessed for all rats in the study 24 h postoperation.

### 2.4. Tissue homogenization, RNA isolation, quantification, and qualification

SC segments were crushed using disposable polypropylene pestles and homogenized with 20-gauge syringe needles. Total RNA was isolated using the RNeasy Plus Mini Kit (QIAGEN, Hilden, Germany). RNase-free DNase I (Thermo Fisher Scientific, MA, USA) was used to remove genomic DNA. Sodium acetate (5M, pH 5.2) was used to remove excess contamination from the DNase I treatment. The purity and concentration of RNA were first assessed spectrophotometrically with a NanoDrop 8000 spectrophotometer (Thermo Fisher Scientific, MA, USA) to exclude impure or low-concentrated samples. RNA quality was checked using the Agilent RNA 6000 Nano Kit on the 2100 Bioanalyzer (Agilent Technologies, CA, USA). RNA quantification was performed by using a Qubit RNA BR Assay Kit on Qubit 2.0 Fluorometer (Invitrogen, MA, USA). Samples with an RNA integrity number (RIN) more than 6 were preserved for library preparation.

### 2.5. Library preparation and RNA sequencing

Prior to library preparation, samples were diluted in 10–12 μL with ultrapure water so that the final concentration of the RNA was at least 400 ng. Cytoplasmic and mitochondrial rRNAs were removed according to the TruSeq Stranded Total RNA Library Prep workflow with Ribo-Zero Gold in the Illumina TruSeq Stranded Total RNA reference guide (San Diego, CA, USA). RNA was fragmented and purified using RNAClean XP beads to select 150–200 bp fragments. After the generation of double-stranded cDNA, the 3’ ends of the cDNAs were adenylated prior to the ligation of unique dual index adapters to protruding thymines. Adapter-ligated DNA fragments were selectively amplified using a T Professional thermocycler (Biometra GmbH, Analytik Jena AG, Göttingen, Germany). The libraries were quantified using the KAPA Library Quant Kit ABI Prism qPCR Mix (Roche, KAPA Biosystems, Cape Town, South Africa) and performing qPCR on a ViiA 7 Real-Time PCR system (Applied Biosystems, Waltham, MA, USA). Library purity and fragment length distribution was assessed using Agilent High Sensitivity DNA Kit on a 2100 Bioanalyzer (Agilent Technologies, CA, USA).

Template DNA libraries were normalized according to Protocol A (standard loading) in Illumina’s NovaSeq 6000 Denature and Dilute Libraries Guide so that each pooled library had a concentration of 2.25 nM, i.e. the final loading concentration was 450 pM. Thereafter, the libraries were mixed into a pool with a final volume of 100 μL. The library pool was denatured by adding 0.2 N NaOH and pipetted into the library tube in the NovaSeq 6000 SP Reagent Kit (300 cycles), and the samples were sequenced on the NovaSeq 6000 sequencing system (Illumina, San Diego, CA, USA).

### 2.6. Quality control, transcript assembly, and differential expression analyses

Conversion of bcl files to fastq files and adaptor trimming was performed using bcl2fastq2 version 2.20. BowTie2 version 2.4.1 was used for alignment and StringTie version 2.1.3 for transcript assembly. Rnor_6.0 (https://www.ensembl.org/Rattus_norvegicus/Info/Index) was used as the reference genome and for transcript assembly. Finally, differential expression analyses were performed using edgeR version 3.8.6.

### 2.7. Identification of differentially expressed transcription factors and transcription factor binding site predictions

Differentially expressed transcription factors (TFs) in our data were identified using the AnimalTFDB version 4.0 database, which provides genome-wide classification and annotation of TFs and cofactors across 183 animal genomes, including *Rattus norvegicus* [38]. To determine the relationship between the identified TFs and the differentially expressed *AQP* genes, TF binding site predictions were also conducted using the AnimalTFDB version 4.0 database [38]. Therefore, the fasta sequences of *Aqp1* (accession: BC090068), *Aqp4* (AF144082), *Aqp9* (AF016406), and *Aqp11* (AB023644) were obtained from the European Nucleotide Archive (ENA) and analyzed for TF binding sites.

### 2.8. Visualization of transcriptome data

Volcano plots were generated with R version 4.0.2 in RStudio version 1.3.1056 using the packages ggplot2 version 3.3.2, dplyr version 1.0.1, and ggrepel version 0.8.2 [39–43]. The heatmap was created by using Heatmapper [44].

### 2.9. Gene set enrichment analysis of differentially expressed genes

Gene set enrichment analysis was performed using the Enrichr tool (https://maayanlab.cloud/Enrichr/) [45]. As part of the gene ontology (GO) analysis, biological processes and molecular functions of differentially expressed genes (DEGs) were investigated, and the WikiPathways database was used for pathway analyses.

## 3. Results

### 3.1. Animal experiments and neurological examination

**Figure 2:**
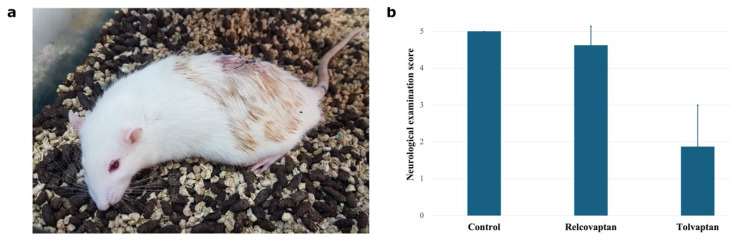
Animal subject and neurological examination scores. (a) Immobile rat after postsurgical treatment with tolvaptan. (b) Bar chart showing Gale’s motor function scores.

Tolvaptan treatment induced bradycardia and caused one death within 24 h (Figure 2a), whereas no mortality occurred in the relcovaptan group. Moreover, neurological scores at 24 h post-tSCI were significantly higher in relcovaptan-treated rats compared to those receiving tolvaptan (Figure 2b).

### 3.2. Differential gene expression analysis and expression of aquaporin genes

**Figure 3:**
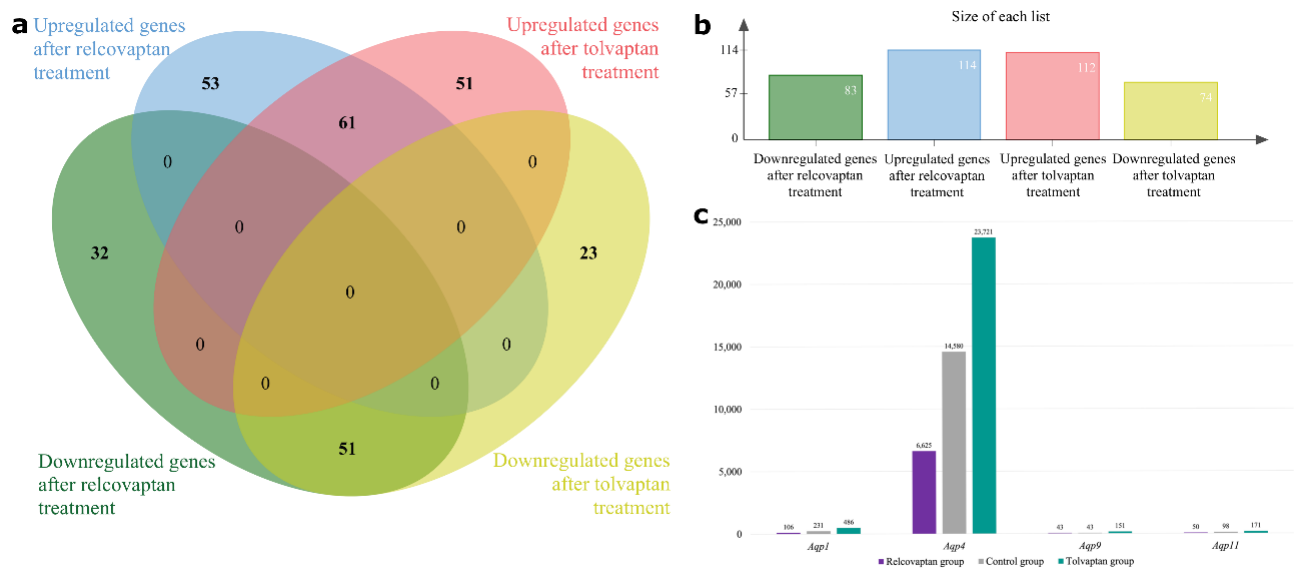
Unique DEGs after treatments and AQP gene expression levels. (a) Venn diagram showing the number of unique DEGs. (b) While there were 114 upregulated genes and 83 downregulated genes in rats treated with relcovaptan, there were 112 upregulated genes and 74 downregulated genes in rats treated with tolvaptan. Generated with jvenn [71]. (c) Mean expression levels of *Aqp1*, *Aqp4*, *Aqp9*, and *Aqp11* in the control and treatment groups.

We found 383 DEGs (271 unique DEGs when deduplicated) with p < 0.05 and fold change (FC) ≤ 0.25 (log_2_FC ≤ −2) for downregulated genes and FC ≥ 4 (log_2_FC > 2) for upregulated genes (Figures 3a and 3b) (Supplementary Data 1 and 2). The 30 DEGs with most extreme FCs (15 upregulated and 15 downregulated) in response to relcovaptan and tolvaptan are listed in Tables 1 and 2, respectively.

Regarding *Aqp* gene expression, only *Aqp1*, *Aqp4*, *Aqp9*, and *Aqp11* were detected in the SCs of control animals. Compared to the control group, relcovaptan treatment resulted in lower expression of these AQPs (equal in the case of *Aqp9*), whereas tolvaptan treatment led to higher expression levels (Figure 3c).

**Figure 4:**
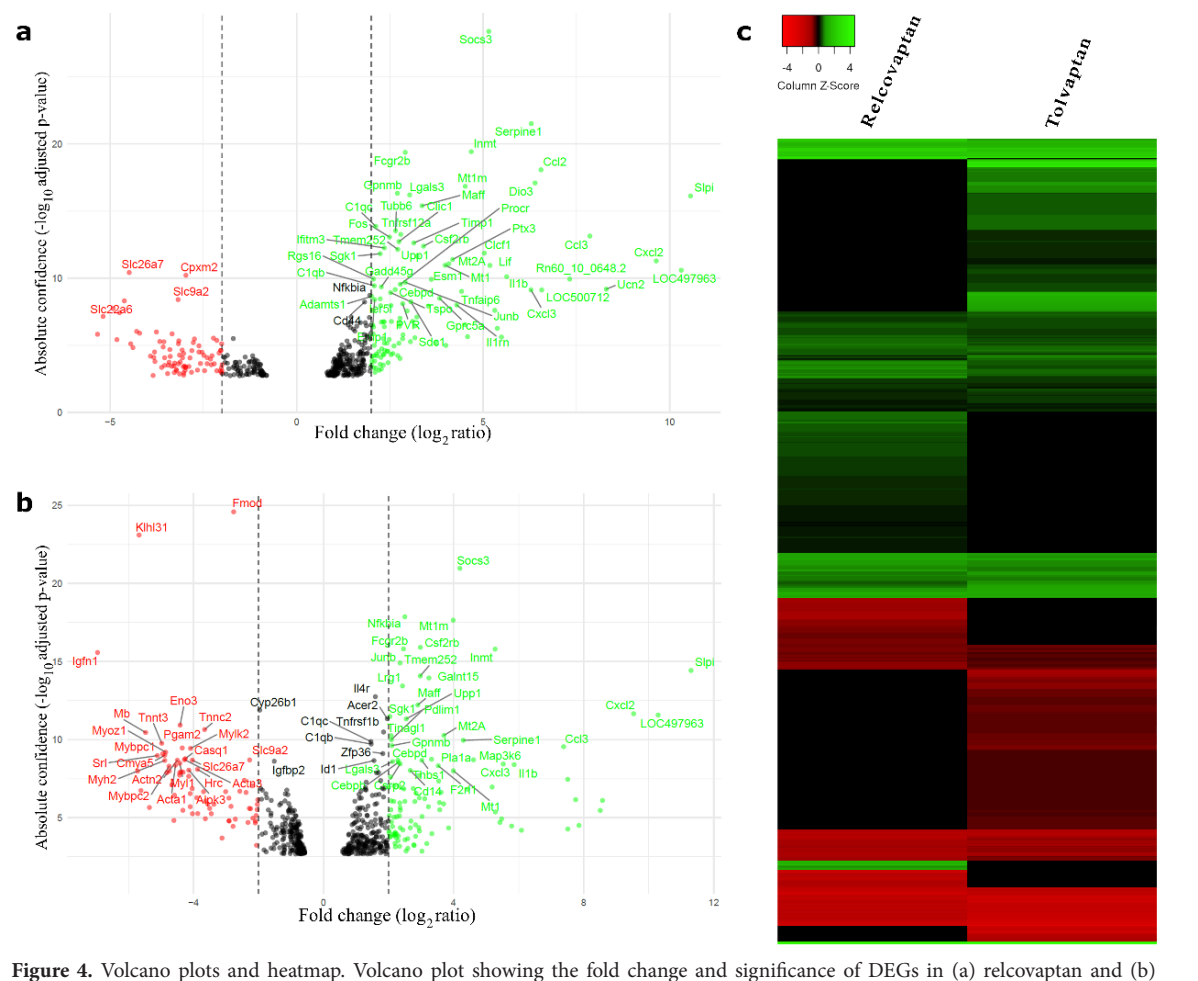
Volcano plots and heatmap. Volcano plot showing the fold change and significance of DEGs in (a) relcovaptan and (b) tolvaptan treatment groups. (c) Heatmap showing upregulated genes in green and downregulated genes in red. Black dots (in volcano plots) and lines (in heatmap) represent genes that are not differentially expressed between each treatment group and the control group.

The volcano plots of the relcovaptan and tolvaptan groups, along with the heatmap, show partially overlapping expression patterns, reflecting the shared impact of tSCI (Figure 4).

### 3.3. Differentially expressed TFs and predicted TF binding sites of differentially expressed aquaporins

The TF genes MAF bZIP transcription factor F (*Maff*), CCAAT/enhancer binding protein beta (*Cebpb*), and CCAAT/enhancer binding protein delta (*Cebpd*) were upregulated in both relcovaptan- and tolvaptan-treated rats, whereas six homeobox 1 (*Six1*) was downregulated in both treatments.

Homeobox B13 (*Hoxb13*) was only differentially expressed after tolvaptan treatment. In contrast, SRY-box transcription factor 7 (*Sox7*), fos proto-oncogene AP-1 transcription factor subunit (*Fos*), pleckstrin (*Plek*), HOP homeobox (*Hopx*), activating transcription factor 3 (*Atf3*), spi-b transcription factor (*Spib*), and recombination activating 1 (*Rag1*) were differentially expressed after relcovaptan treatment (Table S1 in Supplementary Data 3).

Further analysis showed that HOPX, PLEK, and RAG1 do not bind to the predicted TF binding sites of *Aqp1*, *Aqp4*, *Aqp9*, and *Aqp11*; whereas CEBPB, HOXB13, and SOX7 may bind to the predicted TF binding sites, however, with high false discovery rates (q > 1.0) (Tables S2–S5 in Supplementary Data 3). ATF3, CEBPD, FOS, MAFF, SIX1, and SPIB were the only significant TFs with q < 1.0 (Table S6 in Supplementary Data 3). While ATF3 and FOS were upregulated and SPIB was downregulated after relcovaptan treatment, none of these 3 TFs were expressed after tolvaptan treatment, suggesting that their differential regulation may contribute to the distinct AQP gene expression patterns between the treatments.

### 3.4. Gene ontology analysis

**Figure 5:**
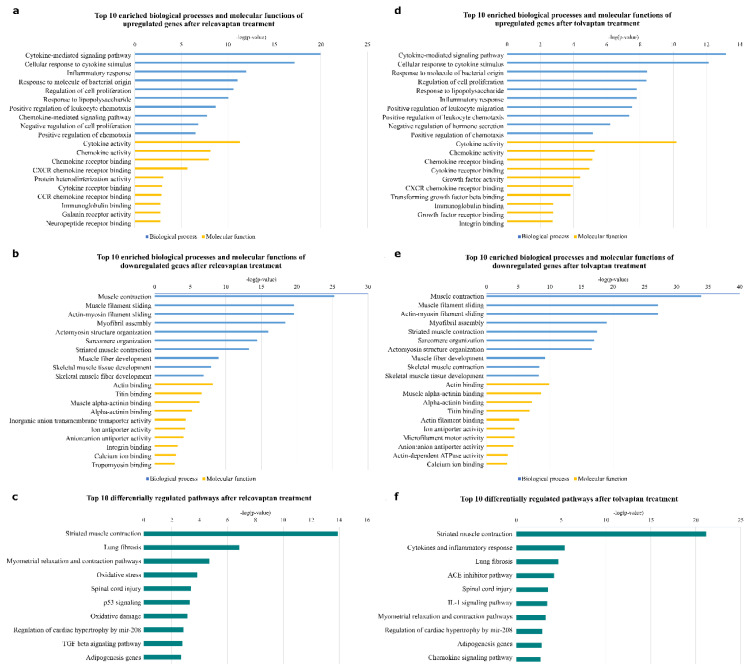
Results of GO and pathway analysis. The top 10 enriched GO terms are presented for (a) upregulated genes and (b) downregulated genes in response to relcovaptan, as well as (c) the top 10 differentially regulated pathways after relcovaptan treatment. Correspondingly, the top 10 enriched GO terms are shown for (d) upregulated genes and (e) downregulated genes in response to tolvaptan, as well as (f) the top 10 differentially regulated pathways after tolvaptan treatment. Blue bars indicate biological processes, and orange bars indicate molecular functions. The degree of enrichment of each GO term and pathway is indicated by −log(p-value) (x-axis).

GO analysis showed that immunological processes were upregulated in both treatment groups, as expected. In contrast, muscle-associated processes such as muscle contraction, muscle filament sliding, and myofibril assembly were downregulated. Similarly, upregulated molecular functions were related to immunological functions and downregulated molecular functions to muscle-associated functions (Figures 5a–5e) (Supplementary Data 4). However, it is noticeable that molecular functions such as protein heterodimerization activity, CCR chemokine receptor binding, galanin receptor activity, and neuropeptide receptor binding were among the top 10 upregulated molecular functions in relcovaptan treatment. On the contrary, growth factor activity, transforming growth factor (TGF) beta binding, growth factor receptor binding, and integrin binding were among the top 10 upregulated molecular functions in tolvaptan treatment. Integrin binding was one of the most downregulated molecular functions in relcovaptan treatment.

### 3.5. Pathway analysis

The results of pathway analysis were similar to some extent in both treatment groups (Supplementary Data 5). Pathways such as striated muscle contraction, lung fibrosis, myometrial relaxation and contraction pathways, spinal cord injury, regulation of cardiac hypertrophy by miR-208, and adipogenesis genes were differentially regulated in both treatments when compared with the control group. Among the top 10 differentially regulated pathways were oxidative stress, p53 signaling, oxidative damage, and TGF beta signaling pathway in relcovaptan treatment; cytokines and inflammatory response, angiotensin-converting enzyme (ACE) inhibitor pathway, interleukin-1 signaling pathway, and chemokine signaling pathway were differentially regulated in tolvaptan treatment (Figures 5c and 5f).

## 4. Discussion

Our results showed that *Aqp1*, *Aqp4*, *Aqp9*, and *Aqp11* were expressed in SC, whereas *Aqp0*, *Aqp2*, *Aqp3*, *Aqp5*, *Aqp6*, *Aqp7*, *Aqp8*, *Aqp10*, and *Aqp12* were not detected.

We report the therapeutic effect of relcovaptan, a nonpeptide V1aR antagonist, for the first time in tSCI. Relcovaptan counteracts the pathophysiological overexpression of *Aqp1*, *Aqp4*, and *Aqp11* in tSCI by downregulating these genes, an effect not observed with tolvaptan. As a result, a favorable outcome regarding edema is reflected in the excellent condition of the relcovaptan-treated rats. Relcovaptan is known to downregulate *Aqp4* after brain injury [46–48]. However, its effect on *Aqp4* expression following tSCI has not previously been investigated. Thus, to the best of our knowledge, this is the first report on the downregulating effect of relcovaptan not only on *Aqp4* but also on *Aqp1* and *Aqp11* expression. The mean expression level of *Aqp9* was identical between the control and relcovaptan groups, and 3.5-fold higher in the tolvaptan group. This finding suggests that *Aqp9* is unlikely to be regulated by V1aR and V2R or via vasopressin, confirming the results of Moeller et al. [49] who previously showed that *Aqp9* is not regulated by V1aR and V2R. Indeed, studies show that *Aqp9* is regulated by systemic insulin, extracellular pH, and hyperosmotic stress due to two hypertonicity response elements in the promoter region of *Aqp9* [50]. Moreover, Tsukaguchi et al. [51] suggested the regulation of *Aqp9* by glucagon and glucocorticoid because of the glucocorticoid-responsive element in the *Aqp9* gene.

Furthermore, GO analysis showed that biological processes related to the immune system such as cytokine-mediated signaling pathway (involving the potential TFs CEBPD and FOS after relcovaptan, and CEBPD after tolvaptan treatment, among other genes), inflammatory response (involving the potential TF FOS after relcovaptan treatment, among other genes), and positive regulation of leukocyte chemotaxis increased in subjects treated with relcovaptan and tolvaptan compared to control subjects. Both drug treatments induced a response to bacteria-derived molecules such as lipopolysaccharide, likely due to intracellular bacterial invasion following disruption of BSCB integrity [52]. Muscle-related biological processes including muscle contraction, muscle filament shift, sarcomere organization, muscle fiber development, and skeletal muscle tissue development and molecular functions such as actin and α-actin binding and titin binding were enriched in both drug administrations as expected due to the flaccid paralysis that develops immediately after trauma [53]. Other symptoms, including muscle fatigue and muscle atrophy encountered after tSCI, could develop through the deterioration in the control of Ca^2+^-release into the sarcoplasmic reticulum. This could be because of changes in Ca^2+^-ATPase enzyme function or concentration, which in turn can lead to the onset of neurodegeneration [54]. This phenomenon is reflected in the GO terms ion antiporter activity, calcium ion binding, and actin-dependent ATPase activity that we detected in the current study. Similarly, immunological functions such as cytokine and chemokine activities—secreted by astrocytes and microglia, leading to the recruitment of proinflammatory cells—were enriched at the GO molecular function level in both drug treatments [54,55]. However, unlike tolvaptan, the top 10 enriched molecular functions in relcovaptan treatment include protein heterodimerization activity (involving the potential TFs ATF3 and FOS, among other genes), CCR chemokine receptor binding, galanin receptor activity, and neuropeptide receptor binding. The enrichment of protein heterodimerization activity and CCR chemokine receptor binding suggests that the immune response in relcovaptan-treated subjects may be regulated by heterodimer-forming chemokines. These chemokines are thought to have therapeutic potential due to their regulatory effect on leukocyte responses and disruption of specific interactions [56]. ATF3, FOS, and MAFF, identified as potential TFs of *Aqp1*, *Aqp4*, *Aqp9*, and *Aqp11*, belong to the ATF, Fos, and Maf subfamilies, respectively, which together with the Jun subfamily form the activator protein-1 (AP-1) family [57–59]. In our study, no member of the Jun subfamily was identified as a potential TF of the *AQP* genes. However, ATF3, FOS, and MAFF, together with JUNB, were found to play a role in many GO biological processes, such as positive regulation of transcription from RNA polymerase II promoter, positive regulation of DNA-templated transcription, and regulation of transcription from RNA polymerase II promoter. Strikingly, galanin (GAL), CCAAT enhancer binding protein beta (CEBPB), serpin family E member 1 (SERPINE1), LIF interleukin 6 family cytokine (LIF), oncostatin M (OSM), toll-like receptor 2 (TLR2), and follistatin-like 3 (FSTL3) were found to co-occur with ATF3, FOS, MAFF, and JUNB in all 3 biological processes, suggesting a potential interplay between these proteins in the regulation of transcription. GO molecular function analysis showed that ATF3 was involved in protein homodimerization activity and, together with FOS, in protein heterodimerization activity.

AP-1 proteins function as homo- or heterodimers with distinct DNA-binding and transcriptional activities. Although mainly activators, they can act as repressors in a context-dependent manner. They regulate processes including the cell cycle, proliferation, differentiation, apoptosis, autophagy, migration, invasion, and lipid synthesis, and are implicated in inflammation, bone development, nervous system function, immune cell regulation, and cancer [59,60]. Our results suggest that ATF3–FOS heterodimers function as transcriptional repressors targeting *Aqp1* and possibly *Aqp9*, causing the downregulation of *Aqp1* and the prevention of *Aqp9* overexpression. Regarding *Aqp9* expression, our GO molecular function results indicate that another possible transcriptional regulator is ATF3 homodimer. ATF3, usually low in resting cells and upregulated by stressors such as oxidative damage and ischemia/reperfusion [61], remained unchanged in traumatized tolvaptan treatment. These findings show that ATF3 upregulation is driven by relcovaptan. Similarly, FOS upregulation and SPIB downregulation are also attributable to relcovaptan. SPIB is a TF that plays a key role in B cell differentiation and function in the immune system [62]. Our prediction analysis for TF binding sites showed that SPIB may bind to *Aqp4*, *Aqp9*, and *Aqp11* and regulate their expression. Furthermore, SPIB downregulation is correlated with the downregulation of *Aqp4*, *Aqp9*, and *Aqp11*, suggesting that SPIB is an activator of these *AQP* genes.

The upregulation of GAL and its receptor activity is notable, as GAL exerts immunoregulatory, neuroprotective, and neuroregenerative effects and regulates osmotic homeostasis by inhibiting vasopressin release [63].

As noted, GAL co-occurs with AP-1 proteins. Given that AP-1 includes ATF3, which is strongly linked to neuronal regrowth and regeneration across vertebrates, a synergistic effect of GAL and ATF3 is possible. Moreover, ATF3 overexpression has been shown to exert a neuroprotective effect on a subtype of retinal ganglion cells after optic nerve crush, suggesting its involvement not only in axon regeneration but also in neuronal survival [57,64].

Signaling pathways like spinal cord injury, lung fibrosis, and adipogenesis genes reflect tSCI-related conditions, including respiratory dysfunction, respiratory muscle fatigue, pleuropulmonary pathology, and adipose accumulation due to impaired physical mobility [53,65]. Similarly, pathways like striated muscle contraction, cytokines and inflammatory response, oxidative stress, IL-1 signaling pathway, p53 signaling, oxidative damage, TGF beta signaling pathway, and chemokine signaling pathway were differentially regulated due to their association with tSCI. For instance, secondary injury-induced reactive oxygen species triggers oxidative stress, altering polyunsaturated membrane lipids (e.g., linoleic and arachidonic acids) and damaging proteins and DNA, thereby causing oxidative damage [66]. As a result of DNA damage, p53 signaling is activated, and neuronal apoptosis occurs. Interestingly, the myometrial relaxation and contraction pathway was differentially regulated by both drugs despite the use of male rats, likely due to their antagonism of the oxytocin receptor in addition to V1aR and V2R [67,68]. The regulation of cardiac hypertrophy by miR-208 pathway was affected by both drugs, as V1aR (targeted by relcovaptan) is linked to cardiac hypertrophy, and V2R (targeted by tolvaptan) may have therapeutic potential in heart failure [69]. Unlike relcovaptan, tolvaptan affected the ACE inhibitor pathway, which is linked to increased immune reactivity and worsened neuronal damage through substance P degradation after brain injury, thereby exacerbating neuronal damage and motor deficits [70]. Since no association between ACE inhibition and the pathophysiologic mechanism of tSCI has been found in the literature, we hypothesize that the inhibition of ACE is due to the effect of tolvaptan.

### Limitations

The primary aim of this study was to compare the effects of relcovaptan and tolvaptan in a tSCI model. For this reason, no sham-operated group was included, in line with the 3Rs principle to minimize animal use. Edema was assessed at the transcriptional level, which provided valuable initial insights into the involvement of AQP genes. Future studies should build on these findings by performing dose-response experiments, ideally in combination with appropriate control and sham groups, to better define the therapeutic window and confirm edema extent and localization through histological and imaging evidence. In addition, the relatively small sample size and limited number of experimental groups reflect the focused design of this comparative study, but larger cohorts will be required to validate and generalize the results. Finally, further research should also explore the regulatory interactions between the identified TFs (ATF3, CEBPD, FOS, MAFF, SIX1, and SPIB) and AQP genes (*Aqp1*, *Aqp4*, *Aqp9*, and *Aqp11*), in order to elucidate potential mechanistic pathways.

## 5. Conclusion

This study shows, for the first time, that relcovaptan, a nonpeptide V1aR antagonist, exerts therapeutic effects in tSCI in adult rats by targeting multiple protective mechanisms. Relcovaptan significantly downregulates *Aqp1*, *Aqp4*, and *Aqp11*, while preventing pathological *Aqp9* upregulation, unlike tolvaptan, which showed no benefit and may worsen neuronal damage by disturbing the degradation of substance P through the inhibition of the ACE pathway, a mechanism known to increase immune reactivity.

Transcriptome analysis showed neuroprotective effects—including anti-apoptotic, anti-inflammatory anti-excitotoxic, immunomodulatory, and regenerative properties—likely mediated by the regulation of the TFs ATF3, FOS, and MAFF (AP-1 family) and SPIB downregulation, a novel potential transcriptional activator of *Aqp4*, *Aqp9*, and *Aqp11*. ATF3–FOS heterodimers may repress *Aqp1*, and ATF3 homodimers may repress *Aqp9*, suggesting new transcriptional regulatory mechanisms.

Functional enrichment analysis indicated involvement in immune response regulation, neuronal survival, and oxidative stress response. Upregulation of GAL and its co-expression with AP-1 proteins indicate a synergistic role in neuroprotection and regeneration.

Overall, relcovaptan modulates *Aqp* gene expression and key transcriptional networks, offering a novel antiedema and neuroprotective approach for tSCI. AP-1 proteins themselves may represent future therapeutic targets.

Further studies are needed to validate the interaction between AP-1 proteins, including ATF3, SPIB, and AQP genes, assess long-term effects, and confirm edema reduction. The GAL–AP-1 co-regulatory axis may also provide new treatment strategies. In addition, continuous research and clinical trials are necessary to validate and translate relcovaptan therapy into routine clinical practice for tSCI as a neuroprotective agent.

## Supplementary Information











## Figures and Tables

**Table 1:** The 15 most downregulated and 15 most upregulated DEGs after relcovaptan treatment.

Downregulated Genes				#	Upregulated Genes			
Ensembl ID	Gene Name	log_10_(p-Value)	log_2_(FC)		Ensembl ID	Gene Name	log_10_(p-Value)	log_2_(FC)
ENSRNOG00000032443	*Lmod3*	−5.817	−5.344	1	ENSRNOG00000046699	*Slpi*	−16.122	10.562
ENSRNOG00000026087	*Igfn1*	−7.146	−5.185	2	ENSRNOG00000057443	*LOC497963*	−10.587	10.308
ENSRNOG00000004398	*Pkhd1l1*	−7.726	−4.923	3	ENSRNOG00000002792	*Cxcl2*	−11.275	9.64
ENSRNOG00000059350	*Ppp1r3a*	−5.417	−4.819	4	ENSRNOG00000020655	*Ucn2*	−9.181	8.295
ENSRNOG00000012609	*Trdn*	−7.433	−4.748	5	ENSRNOG00000011205	*Ccl3*	−13.133	7.863
ENSRNOG00000018215	*Slc22a6*	−8.306	−4.618	6	ENSRNOG00000062228	*Rn60_10_0648.2*	−9.929	7.319
ENSRNOG00000006096	*Slc26a7*	−10.418	−4.486	7	ENSRNOG00000010478	*LOC500712*	−9.115	6.568
ENSRNOG00000024330	*Ngp*	−5.106	−4.447	8	ENSRNOG00000007159	*Ccl2*	−18.07	6.548
ENSRNOG00000008310	*Mpo*	−4.826	−4.385	9	ENSRNOG00000052017	*Dio3*	−17.091	6.39
ENSRNOG00000057404	*Slc47a1*	−6.033	−4.289	10	ENSRNOG00000001414	*Serpine1*	−21.506	6.285
ENSRNOG00000006224	*Klhl31*	−5.904	−4.212	11	ENSRNOG00000028043	*Cxcl3*	−9.131	6.275
ENSRNOG00000021200	*Hfe2*	−4.187	−3.918	12	ENSRNOG00000004649	*Il1b*	−10.112	5.628
ENSRNOG00000025757	*Myh6*	−2.75	−3.847	13	ENSRNOG00000014378	*Il1r2*	−5.609	5.491
ENSRNOG00000049942	*RGD1564899*	−4.085	−3.832	14	ENSRNOG00000002802	*Cxcl1*	−6.261	5.379
ENSRNOG00000022777	*Six1*	−4.495	−3.812	15	ENSRNOG00000009919	*Acod1*	−7.598	5.305

**Table 2:** The 15 most downregulated and 15 most upregulated DEGs after tolvaptan treatment.

Downregulated Genes				#	Upregulated Genes			
Ensembl ID	Gene Name	log_10_(p-Value)	log_2_(FC)		Ensembl ID	Gene Name	log_10_(p-Value)	log_2_(FC)
ENSRNOG00000026087	*Igfn1*	−15.577	−6.952	1	ENSRNOG00000046699	*Slpi*	−14.425	11.303
ENSRNOG00000012609	*Trdn*	−7.99	−5.72	2	ENSRNOG00000057443	*LOC497963*	−11.582	10.29
ENSRNOG00000006224	*Klhl31*	−23.097	−5.674	3	ENSRNOG00000002792	*Cxcl2*	−11.657	9.536
ENSRNOG00000059350	*Ppp1r3a*	−6.741	−5.617	4	ENSRNOG00000008525	*Csf3*	−6.106	8.579
ENSRNOG00000004583	*Mb*	−10.46	−5.472	5	ENSRNOG00000019500	*Cyp1a1*	−5.471	8.511
ENSRNOG00000021200	*Hfe2*	−5.657	−5.356	6	ENSRNOG00000017386	*Il11*	−4.507	7.857
ENSRNOG00000005269	*Srl*	−8.981	−5.111	7	ENSRNOG00000058238	*Rn50_20_0046.8*	−6.159	7.754
ENSRNOG00000020332	*Tnnt3*	−9.768	−4.978	8	ENSRNOG00000030387	*Kng1*	−4.282	7.511
ENSRNOG00000056493	*Mybpc1*	−9.09	−4.95	9	ENSRNOG00000055889	*AABR07030901.1*	−7.463	7.508
ENSRNOG00000023803	*Cmya5*	−9.003	−4.885	10	ENSRNOG00000011205	*Ccl3*	−9.548	7.384
ENSRNOG00000049695	*Myh2*	−8.668	−4.884	11	ENSRNOG00000062228	*Rn60_10_0648.2*	−4.202	6.087
ENSRNOG00000040122	*Myoz1*	−9.201	−4.855	12	ENSRNOG00000004649	*Il1b*	−8.401	5.862
ENSRNOG00000007999	*Abra*	−7.894	−4.818	13	ENSRNOG00000029386	*RT1-*	−4.466	5.785
ENSRNOG00000019627	*Mybpc2*	−8.303	−4.751	14	ENSRNOG00000028043	*Cxcl3*	−8.436	5.527
ENSRNOG00000049942	*RGD1564899*	−7.985	−4.745	15	ENSRNOG00000014378	*Il1r2*	−4.934	5.477
