# Supplementary material for: Relcovaptan: a promising therapeutic agent in traumatic spinal cord injury that acts by modulating newly identified transcriptional regulators of aquaporins compared to tolvaptan
Source: Turk J Med Sci. 2025 Sep 22;55(6):1394–407. doi: 10.55730/1300-0144.6097 (PMC12779027; doi:10.55730/1300-0144.6097)
Supplement: Supplementary file 1 [file MedSci_55-6-1394_Supplementary-Data-1.pdf]

Supplementary Data 1: Differentially Expressed Genes After Relcovaptan Treatment

Table S1. Upregulated Genes After Relcovaptan Treatment. 114 Upregulated Genes After Relcovaptan Treatment. Genes are ordered from most upregulated to least upregulated genes. The expression of genes marked with an asterisk (*) was also upregulated in rats treated with Tolvaptan.

Ensembl Stable ID	Gene Name	log_{10}(p-value)	log_2(FC)
ENSRNOG00000046699*	Slpi	-16.122	10.562
ENSRNOG00000057443*	LOC497963	-10.587	10.308
ENSRNOG00000002792*	Cxcl2	-11.275	9.64
ENSRNOG00000020655	Ucn2	-9.181	8.295
ENSRNOG00000011205*	Ccl3	-13.133	7.863
ENSRNOG00000062228*	Rn60_10_0648.2	-9.929	7.319
ENSRNOG00000010478*	LOC500712	-9.115	6.568
ENSRNOG00000007159*	Ccl2	-18.07	6.548
ENSRNOG00000052017*	Dio3	-17.091	6.39
ENSRNOG00000001414*	Serpine1	-21.506	6.285
ENSRNOG00000028043*	Cxcl3	-9.131	6.275
ENSRNOG00000004649*	Il1b	-10.112	5.628
ENSRNOG00000014378*	Il1r2	-5.609	5.491
ENSRNOG00000002802*	Cxcl1	-6.261	5.379
ENSRNOG00000009919*	Acod1	-7.598	5.305
ENSRNOG00000007002*	Lif	-10.947	5.183
ENSRNOG00000002946*	Socs3	-28.384	5.154
ENSRNOG00000018752*	Clcf1	-11.86	5.033
ENSRNOG00000011250*	Inmt	-19.42	4.684
ENSRNOG00000013973	Lcn2	-5.643	4.578
ENSRNOG00000028841*	Mt1m	-16.842	4.517
ENSRNOG00000024390*	Osm	-6.544	4.507
ENSRNOG00000050792	Tnfaip6	-9.02	4.423
ENSRNOG00000005871*	Il1rn	-8.003	4.289
ENSRNOG00000012280	Ptx3	-11.387	4.18

ENSRNOG00000043098*	Mt2A	-11.074	4.076
ENSRNOG00000022242	Cxcl9	-4.989	3.999
ENSRNOG00000038047*	Mt1	-10.958	3.975
ENSRNOG00000008412*	Gprc5a	-8.5	3.828
ENSRNOG00000008942	RGD1304963	-5.156	3.753
ENSRNOG00000010797*	Esm1	-9.912	3.607
ENSRNOG00000057153*	Pla1a	-7.922	3.525
ENSRNOG00000000187*	Csf2rb	-12.382	3.402
ENSRNOG00000012886*	Maff	-15.386	3.364
ENSRNOG00000004972*	Upp1	-11.632	3.231
ENSRNOG00000027811*	Lilrb4	-7.102	3.216
ENSRNOG00000017819*	Cd14	-6.441	3.174
ENSRNOG00000045829*	Thbs1	-5.557	3.173
ENSRNOG00000010208	Timp1	-12.618	3.142
ENSRNOG00000000239	Ccl7	-6.778	3.109
ENSRNOG00000059947*	Sdc1	-8.24	3.059
ENSRNOG00000016413*	Pstpip1	-5.27	3.046
ENSRNOG00000010645*	Lgals3	-16.204	3.027
ENSRNOG00000036834	Gpr84	-7.556	2.959
ENSRNOG00000042838*	Junb	-9.688	2.911
ENSRNOG00000046452*	Fcgr2b	-19.369	2.906
ENSRNOG00000018054	F2rl2	-5.778	2.853
ENSRNOG00000010181*	Clec4d	-5.477	2.844
ENSRNOG00000019202*	PVR	-8.1	2.835
ENSRNOG00000003546	Tnfrsf12a	-13.257	2.788
ENSRNOG00000019330*	Procr	-9.539	2.777
ENSRNOG00000012049	Sox7	-6.671	2.774
ENSRNOG00000006320*	Ptges	-7.011	2.774
ENSRNOG00000026653*	Hcar2	-4.024	2.767
ENSRNOG00000023546	Hspb1	-6.423	2.763
ENSRNOG00000017484*	Gja5	-5.145	2.742
ENSRNOG00000029682	Clic1	-12.715	2.74

ENSRNOG00000047606*	Bcl2a1	-4.229	2.713
ENSRNOG00000025476*	Tmem252	-12.144	2.705
ENSRNOG00000008816*	Gpnmb	-16.322	2.7
ENSRNOG00000018371	Tubb6	-13.534	2.649
ENSRNOG00000050869*	Cebpd	-9.148	2.641
ENSRNOG00000047300*	Bdkrb2	-4.947	2.613
ENSRNOG00000056219	Olr1	-4.382	2.583
ENSRNOG00000021674	Ifitm7	-6.774	2.543
ENSRNOG00000026306	Clec5a	-4.31	2.529
ENSRNOG00000012494	Kctd14	-7.958	2.528
ENSRNOG00000010549	Tspo	-8.923	2.522
ENSRNOG00000008015	Fos	-13.062	2.492
ENSRNOG00000005214	Plek	-5.427	2.482
ENSRNOG00000043486*	Tnfrsf26	-3.7	2.447
ENSRNOG00000047977	LOC100910163	-4.564	2.438
ENSRNOG00000014117	Hmox1	-4.547	2.431
ENSRNOG00000019106	Rps17	-5.15	2.421
ENSRNOG00000004273*	Ifitm1	-4.615	2.356
ENSRNOG00000000521*	Cdkn1a	-6.751	2.352
ENSRNOG00000009822	Tlr2	-6.379	2.351
ENSRNOG00000015078	Ifitm3	-12.245	2.349
ENSRNOG00000033192	Osmr	-5.166	2.347
ENSRNOG00000015156	Gal	-4.324	2.341
ENSRNOG00000016575	Tnfrsf1b	-4.305	2.323
ENSRNOG00000022839*	Ifit3	-6.753	2.321
ENSRNOG00000014838	Glipr2	-3.519	2.32
ENSRNOG00000015599*	Mall	-5.503	2.32
ENSRNOG00000055021*	U6atac	-4.74	2.28
ENSRNOG00000013090	Gadd45g	-9.348	2.272
ENSRNOG00000016166*	Pdlim1	-6.722	2.271
ENSRNOG00000022565	Lrrc25	-3.23	2.269
ENSRNOG00000057347*	Cebpb	-4.102	2.262

ENSRNOG00000008215	Trim47	-7.95	2.249
ENSRNOG00000005731	Birc3	-3.576	2.244
ENSRNOG00000024846	Ier5l	-8.462	2.238
ENSRNOG00000011815*	Sgk1	-11.819	2.235
ENSRNOG00000012094*	Ltbp2	-3.138	2.211
ENSRNOG00000020991	Ms4a6a	-4.521	2.204
ENSRNOG00000024689	Hopx	-4.211	2.15
ENSRNOG00000061910	Igfbp3	-4.334	2.133
ENSRNOG00000051895*	7SK	-3.289	2.127
ENSRNOG00000012804	C1qc	-13.857	2.12
ENSRNOG00000006019	G0s2	-2.989	2.104
ENSRNOG00000029341	LOC100909911	-3.806	2.094
ENSRNOG00000008676	Emp1	-8.403	2.09
ENSRNOG00000012749	C1qb	-9.429	2.082
ENSRNOG00000009311*	Fstl3	-4.988	2.078
ENSRNOG00000003745	Atf3	-4.224	2.065
ENSRNOG00000006940	Ncf4	-3.329	2.056
ENSRNOG00000049918*	Lrg1	-6.362	2.056
ENSRNOG00000027024	Rgs16	-9.934	2.055
ENSRNOG00000004100	Trib1	-5.845	2.049
ENSRNOG00000014961	Pdpn	-7.588	2.046
ENSRNOG00000000906	Medag	-6.505	2.037
ENSRNOG00000031163	Nfkbiz	-3.831	2.022
ENSRNOG00000057829	SCARNA1	-4.154	2.018
ENSRNOG00000001607	Adamts1	-8.637	2.011

Table S2. Downregulated Genes After Relcovaptan Treatment. 83 Downregulated Genes After Relcovaptan Treatment. Genes are ordered from most downregulated to least downregulated genes. The expression of genes marked with an asterisk (*) was also downregulated in rats treated with Tolvaptan.

Ensembl Stable ID	Gene Name	log_{10}(p-value)	log_2(FC)
ENSRNOG00000032443	Lmod3	-5.817	-5.344
ENSRNOG00000026087*	Igfn1	-7.146	-5.185
ENSRNOG00000004398*	Pkhd1l1	-7.726	-4.923
ENSRNOG00000059350*	Ppp1r3a	-5.417	-4.819
ENSRNOG00000012609*	Trdn	-7.433	-4.748
ENSRNOG00000018215*	Slc22a6	-8.306	-4.618
ENSRNOG00000006096*	Slc26a7	-10.418	-4.486
ENSRNOG00000024330	Ngp	-5.106	-4.447
ENSRNOG00000008310	Mpo	-4.826	-4.385
ENSRNOG00000057404*	Slc47a1	-6.033	-4.289
ENSRNOG00000006224*	Klhl31	-5.904	-4.212
ENSRNOG00000021200*	Hfe2	-4.187	-3.918
ENSRNOG00000025757*	Myh6	-2.75	-3.847
ENSRNOG00000049942*	RGD1564899	-4.085	-3.832
ENSRNOG00000022777*	Six1	-4.495	-3.812
ENSRNOG00000005269*	Srl	-3.789	-3.797
ENSRNOG00000008394	Prg2	-5.991	-3.764
ENSRNOG00000016714*	Nrap	-5.277	-3.671
ENSRNOG00000056493*	Mybpc1	-3.351	-3.615
ENSRNOG00000006930*	Casq1	-4.878	-3.578
ENSRNOG00000028707	Defa7	-5.264	-3.556
ENSRNOG00000049695*	Myh2	-3.496	-3.544
ENSRNOG00000023803*	Cmya5	-4.065	-3.544
ENSRNOG00000016151*	Ankrd23	-2.89	-3.455
ENSRNOG00000007461*	Klhl41	-3.522	-3.444
ENSRNOG0000006783*	Neb	-3.736	-3.422

ENSRNOG00000020557*	Ryr1	-4.682	-3.4
ENSRNOG00000020719*	Hrc	-4.173	-3.388
ENSRNOG00000020733	Camp	-3.438	-3.352
ENSRNOG00000034190	Ighm	-5.847	-3.344
ENSRNOG00000013262*	Myl1	-3.472	-3.291
ENSRNOG00000019627*	Mybpc2	-2.971	-3.276
ENSRNOG00000008235*	Mylk2	-3.668	-3.27
ENSRNOG00000011659*	Alpk3	-3.196	-3.229
ENSRNOG00000058068*	Obscn	-3.101	-3.22
ENSRNOG00000015567*	Slc9a2	-8.395	-3.177
ENSRNOG00000018822	Slc5a5	-5.123	-3.17
ENSRNOG00000017786*	Acta1	-3.615	-3.169
ENSRNOG00000008478	Mmp13	-5.334	-3.162
ENSRNOG00000019745*	Actn3	-4.269	-3.137
ENSRNOG00000017833*	Actn2	-2.869	-3.135
ENSRNOG00000020332*	Tnnt3	-3.759	-3.123
ENSRNOG00000015157*	Smtnl2	-4.639	-3.065
ENSRNOG00000004630	Rag1	-4.068	-3.055
ENSRNOG00000060021*	Txlnb	-3.408	-3.044
ENSRNOG00000004078*	Eno3	-4.388	-3.034
ENSRNOG00000040122*	Myoz1	-2.943	-3.033
ENSRNOG00000022637*	AABR07052585.1	-2.779	-3.032
ENSRNOG00000016837*	Ckm	-3.448	-3.012
ENSRNOG00000034258*	Xirp2	-3.127	-2.986
ENSRNOG00000048402	Igh-6	-3.341	-2.98
ENSRNOG00000015902	Cpxm2	-10.208	-2.971
ENSRNOG00000046763*	Adssl1	-3.377	-2.966
ENSRNOG00000004327*	Ddc	-3.422	-2.89
ENSRNOG00000002158	Ibsp	-4.056	-2.861
ENSRNOG00000020276*	Tnni2	-2.986	-2.821

ENSRNOG00000038135	RatNP-3b	-4.604	-2.809
ENSRNOG00000008356	Myo5c	-5.197	-2.771
ENSRNOG00000027730	Nxpe1	-5.474	-2.741
ENSRNOG00000012134*	Scn4a	-3.145	-2.656
ENSRNOG00000015155*	Tnnc2	-4.498	-2.619
ENSRNOG00000003183*	Fmod	-4.962	-2.615
ENSRNOG00000011754	Myom2	-3.64	-2.584
ENSRNOG00000010666	Wisp2	-4.477	-2.538
ENSRNOG00000019660	Spib	-4.248	-2.489
ENSRNOG00000049829	AABR07060872.1	-3.106	-2.469
ENSRNOG00000045683*	LOC102553715	-4.079	-2.402
ENSRNOG00000025670*	Shisa3	-3.629	-2.396
ENSRNOG00000028627	Hmcn1	-3.967	-2.311
ENSRNOG00000012303*	Apobec2	-3.545	-2.292
ENSRNOG00000002382*	LOC102553715	-4.222	-2.267
ENSRNOG00000010840	Adamtsl3	-5.529	-2.225
ENSRNOG00000011750*	Fam180a	-3.618	-2.208
ENSRNOG00000020951	Slc4a1	-5.363	-2.138
ENSRNOG00000020165	Ahsp	-3.1	-2.135
ENSRNOG00000025198	Gas2l3	-4.423	-2.082
ENSRNOG00000029911	Cilp	-3.09	-2.045
ENSRNOG00000036827	Ppp1r1a	-4.612	-2.044
ENSRNOG00000049642	Smim5	-4.618	-2.03
ENSRNOG00000054768	AABR07050487.1	-2.962	-2.018
ENSRNOG00000052925	NEWGENE_621351	-3.306	-2.015
ENSRNOG00000009227	Aplnr	-5.111	-2.014
ENSRNOG00000003781	Atp10b	-3.545	-2
