# Supplementary material for: Relcovaptan: a promising therapeutic agent in traumatic spinal cord injury that acts by modulating newly identified transcriptional regulators of aquaporins compared to tolvaptan
Source: Turk J Med Sci. 2025 Sep 22;55(6):1394–407. doi: 10.55730/1300-0144.6097 (PMC12779027; doi:10.55730/1300-0144.6097)
Supplement: Supplementary file 2 [file MedSci_55-6-1394_Supplementary-Data-2.pdf]

Supplementary Data 2: Differentially Expressed Genes After Tolvaptan Treatment

Table S1. Upregulated Genes After Tolvaptan Treatment. 112 Upregulated Genes After Tolvaptan Treatment. Genes are ordered from most upregulated to least upregulated genes. The expression of genes marked with an asterisk (*) was also upregulated in rats treated with Relcovaptan.

Ensembl Stable ID	Gene Name	$\log_{10}$(p-value)	$\log_2$(FC)
ENSRNOG00000046699*	Slpi	-14.425	11.303
ENSRNOG00000057443*	LOC497963	-11.582	10.29
ENSRNOG00000002792*	Cxcl2	-11.657	9.536
ENSRNOG00000008525	Csf3	-6.106	8.579
ENSRNOG00000019500	Cyp1a1	-5.471	8.511
ENSRNOG00000017386	Il11	-4.507	7.857
ENSRNOG00000058238	Rn50_20_0046.8	-6.159	7.754
ENSRNOG00000030387	Kng1	-4.282	7.511
ENSRNOG00000055889	AABR07030901.1	-7.463	7.508
ENSRNOG00000011205*	Ccl3	-9.548	7.384
ENSRNOG00000062228*	Rn60_10_0648.2	-4.202	6.087
ENSRNOG00000004649*	Il1b	-8.401	5.862
ENSRNOG00000029386	RT1-N2	-4.466	5.785
ENSRNOG00000028043*	Cxcl3	-8.436	5.527
ENSRNOG00000014378*	Il1r2	-4.934	5.477
ENSRNOG00000009919*	Acod1	-4.696	5.424
ENSRNOG00000002802*	Cxcl1	-5.368	5.284
ENSRNOG00000011250*	Inmt	-15.8	5.276
ENSRNOG00000007159*	Ccl2	-6.965	5.185
ENSRNOG00000008936	Map3k6	-8.712	4.613
ENSRNOG00000052017*	Dio3	-6.827	4.426
ENSRNOG00000001414*	Serpine1	-9.959	4.297
ENSRNOG00000002946*	Socs3	-20.97	4.193
ENSRNOG00000038047*	Mt1	-8.007	3.993
ENSRNOG00000028841*	Mt1m	-17.646	3.989
ENSRNOG00000020136	Tgm1	-4.345	3.845

ENSRNOG00000043098*	Mt2A	-10.268	3.7
ENSRNOG00000024390*	Osm	-5.888	3.694
ENSRNOG00000059272	U5	-6.626	3.614
ENSRNOG00000018752*	Clcf1	-5.511	3.574
ENSRNOG00000007491	Hoxb13	-5.971	3.566
ENSRNOG00000005871*	Il1rn	-7.353	3.542
ENSRNOG00000018003	F2rl1	-8.328	3.528
ENSRNOG00000056651	LOC103690068	-3.978	3.331
ENSRNOG00000057153*	Pla1a	-8.75	3.322
ENSRNOG00000019718	Galnt15	-13.948	3.242
ENSRNOG00000038202	Calml4	-5.711	3.226
ENSRNOG00000022721	Clec18a	-3.347	3.175
ENSRNOG00000008412*	Gprc5a	-6.218	3.11
ENSRNOG00000057562	U5	-5.084	3.029
ENSRNOG00000061999	Rn60_12_0121.3	-2.855	3.026
ENSRNOG00000045829*	Thbs1	-8.682	3.022
ENSRNOG00000000187*	Csf2rb	-15.909	2.979
ENSRNOG00000025476*	Tmem252	-14.077	2.975
ENSRNOG00000000836	Ltb	-4.153	2.949
ENSRNOG00000012094*	Ltbp2	-6.272	2.944
ENSRNOG00000019330*	Procr	-7.709	2.937
ENSRNOG00000010181*	Clec4d	-4.367	2.931
ENSRNOG00000012886*	Maff	-12.223	2.899
ENSRNOG00000009311*	Fstl3	-6.565	2.844
ENSRNOG00000048071	LOC100911776	-3.808	2.83
ENSRNOG00000017484*	Gja5	-4.552	2.827
ENSRNOG00000023465	LOC500300	-6.038	2.808
ENSRNOG00000025860	Drc7	-3.216	2.787
ENSRNOG00000010478*	LOC500712	-3.27	2.776
ENSRNOG00000000521*	Cdkn1a	-6.877	2.758
ENSRNOG00000057568	SNORA84	-4.791	2.687
ENSRNOG00000034772	5S_rRNA	-4.363	2.67

ENSRNOG00000017819*	Cd14	-8.033	2.667
ENSRNOG00000017897	Adam8	-3.038	2.59
ENSRNOG00000051969	SNORA29	-3.657	2.589
ENSRNOG00000004972*	Upp1	-11.328	2.543
ENSRNOG00000027811*	Lilrb4	-2.867	2.538
ENSRNOG00000007002*	Lif	-3.585	2.505
ENSRNOG00000025332	Cd109	-6.048	2.502
ENSRNOG00000007390	Nfkbia	-17.866	2.501
ENSRNOG00000015599*	Mall	-6.854	2.471
ENSRNOG00000046452*	Fcgr2b	-15.812	2.457
ENSRNOG00000028077	Cfap44	-4.215	2.44
ENSRNOG00000049918*	Lrg1	-13.438	2.428
ENSRNOG00000010797*	Esm1	-5.477	2.363
ENSRNOG00000032539	Spag8	-4.358	2.36
ENSRNOG00000004273*	Ifitm1	-5.648	2.356
ENSRNOG00000003772	Csrp2	-8.45	2.356
ENSRNOG00000042838*	Junb	-14.914	2.353
ENSRNOG00000028108	Cytl1	-5.952	2.345
ENSRNOG00000015159	Slc9a3	-3.183	2.338
ENSRNOG00000059947*	Sdc1	-5.123	2.337
ENSRNOG00000022839*	Ifit3	-7.029	2.32
ENSRNOG00000016413*	Pstpip1	-3.827	2.303
ENSRNOG00000057347*	Cebpb	-8.553	2.303
ENSRNOG00000009912	Fgr	-3.542	2.289
ENSRNOG00000050869*	Cebpd	-8.677	2.285
ENSRNOG00000026653*	Hcar2	-5.074	2.256
ENSRNOG00000027935	Lrrc34	-3.954	2.248
ENSRNOG00000059401	Ccdc40	-3.74	2.233
ENSRNOG00000047471	LOC103689994	-3.137	2.21
ENSRNOG00000014269	Cxcr2	-4.068	2.205
ENSRNOG00000003895	Rgs1	-4.001	2.199
ENSRNOG00000061429	Rph3al	-3.361	2.198

ENSRNOG00000043486*	Tnfrsf26	-3.91	2.191
ENSRNOG00000019202*	PVR	-5.036	2.172
ENSRNOG00000060376	LOC100910237	-3.847	2.171
ENSRNOG00000009348	Nos3	-7.054	2.17
ENSRNOG00000051895*	7SK	-6.048	2.161
ENSRNOG00000047606*	Bcl2a1	-3.952	2.148
ENSRNOG00000010645*	Lgals3	-8.599	2.123
ENSRNOG00000017686	Pi15	-4.435	2.12
ENSRNOG00000008816*	Gpnmb	-9.617	2.102
ENSRNOG00000018792	Tekt4	-4.731	2.088
ENSRNOG00000016166*	Pdlim1	-9.999	2.085
ENSRNOG00000013179	Tinagl1	-10.258	2.082
ENSRNOG00000006320*	Ptges	-6.081	2.079
ENSRNOG00000003259	C1qtnf1	-5.508	2.072
ENSRNOG00000012450	Dynlrb2	-3.786	2.058
ENSRNOG00000055021*	U6atac	-7.607	2.058
ENSRNOG00000017473	Ttc25	-3.778	2.044
ENSRNOG00000011815*	Sgk1	-11.494	2.039
ENSRNOG00000047300*	Bdkrb2	-5.143	2.023
ENSRNOG00000055714	Ak7	-4.021	2.018
ENSRNOG00000032807	Rn60_X_0773.5	-4.148	2.016
ENSRNOG00000054360	Tspan11	-6.955	2

Table S2. Downregulated Genes After Tolvaptan Treatment. 74 Downregulated Genes After Tolvaptan Treatment. Genes are ordered from most downregulated to least downregulated genes. The expression of genes marked with an asterisk (*) was also downregulated in rats treated with Relcovaptan.

Ensembl Stable ID	Gene Name	$\log_{10}$(p-value)	$\log_2$(FC)
ENSRNOG00000026087*	Igfn1	-15.577	-6.952
ENSRNOG00000012609*	Trdn	-7.99	-5.72
ENSRNOG00000006224*	Klhl31	-23.097	-5.674
ENSRNOG00000059350*	Ppp1r3a	-6.741	-5.617
ENSRNOG00000004583	Mb	-10.46	-5.472
ENSRNOG00000021200*	Hfe2	-5.657	-5.356
ENSRNOG00000005269*	Srl	-8.981	-5.111
ENSRNOG00000020332*	Tnnt3	-9.768	-4.978
ENSRNOG00000056493*	Mybpc1	-9.09	-4.95
ENSRNOG00000023803*	Cmya5	-9.003	-4.885
ENSRNOG00000049695*	Myh2	-8.668	-4.884
ENSRNOG00000040122*	Myoz1	-9.201	-4.855
ENSRNOG00000007999	Abra	-7.894	-4.818
ENSRNOG00000019627*	Mybpc2	-8.303	-4.751
ENSRNOG00000049942*	RGD1564899	-7.985	-4.745
ENSRNOG00000023227	AABR07052585.2	-7.11	-4.666
ENSRNOG00000016151*	Ankrd23	-4.834	-4.603
ENSRNOG00000020276*	Tnni2	-6.436	-4.585
ENSRNOG00000017786*	Acta1	-8.371	-4.554
ENSRNOG00000017833*	Actn2	-8.678	-4.497
ENSRNOG00000058068*	Obscn	-7.731	-4.428
ENSRNOG00000057701	Myom1	-7.89	-4.426
ENSRNOG00000013262*	Myl1	-8.492	-4.422
ENSRNOG00000004078*	Eno3	-10.929	-4.412
ENSRNOG00000016837*	Ckm	-7.962	-4.377
ENSRNOG00000013532	Pgam2	-9.466	-4.347
ENSRNOG00000017645	Mylpf	-5.483	-4.327

ENSRNOG00000006783*	Neb	-7.908	-4.319
ENSRNOG00000019745*	Actn3	-8.719	-4.279
ENSRNOG00000006930*	Casq1	-8.78	-4.253
ENSRNOG00000022637*	AABR07052585.1	-7.656	-4.146
ENSRNOG00000011659*	Alpk3	-8.051	-4.145
ENSRNOG00000020557*	Ryr1	-6.546	-4.102
ENSRNOG00000000383	Mypn	-5.714	-4.1
ENSRNOG00000008235*	Mylk2	-9.443	-4.092
ENSRNOG00000046763*	Adssl1	-6.867	-4.041
ENSRNOG00000006096*	Slc26a7	-8.689	-4.036
ENSRNOG00000016983	Myh7	-5.262	-4.033
ENSRNOG00000034258*	Xirp2	-6.284	-3.901
ENSRNOG00000020719*	Hrc	-8.103	-3.879
ENSRNOG00000025757*	Myh6	-5.274	-3.869
ENSRNOG00000057404*	Slc47a1	-6.667	-3.691
ENSRNOG00000015155*	Tnnc2	-10.646	-3.659
ENSRNOG00000018215*	Slc22a6	-4.943	-3.636
ENSRNOG00000007461*	Klhl41	-6.082	-3.623
ENSRNOG00000004398*	Pkhd1l1	-5.884	-3.567
ENSRNOG00000048430	Myo18b	-5.601	-3.503
ENSRNOG00000016714*	Nrap	-5.26	-3.453
ENSRNOG00000010079	Car3	-5.855	-3.375
ENSRNOG00000032951	Jsrp1	-3.697	-3.123
ENSRNOG00000016731	Tpm2	-6.666	-3.012
ENSRNOG00000015157*	Smtnl2	-6.255	-2.911
ENSRNOG00000022777*	Six1	-4.806	-2.911
ENSRNOG00000012134*	Scn4a	-4.786	-2.903
ENSRNOG00000012303*	Apobec2	-5.731	-2.847
ENSRNOG00000060021*	Txlnb	-4.446	-2.784
ENSRNOG00000003183*	Fmod	-24.59	-2.764
ENSRNOG00000019810	Des	-4.909	-2.714
ENSRNOG00000012448	Chrnb3	-6.702	-2.575

ENSRNOG00000045683*	LOC102553715	-7.404	-2.436
ENSRNOG00000018086	Slc22a8	-6.624	-2.384
ENSRNOG00000051619	Asb2	-4.695	-2.323
ENSRNOG00000025670*	Shisa3	-5.601	-2.283
ENSRNOG00000015567*	Slc9a2	-8.7	-2.274
ENSRNOG00000004327*	Ddc	-5.582	-2.246
ENSRNOG00000014166	Smoc2	-7.045	-2.193
ENSRNOG00000010266	Cd180	-4.971	-2.176
ENSRNOG00000019184	Npr3	-4.668	-2.101
ENSRNOG00000002382*	LOC102553715	-4.787	-2.092
ENSRNOG00000030763	Dpp4	-5.143	-2.074
ENSRNOG00000019851	Cox6a2	-3.239	-2.062
ENSRNOG00000046258	B3gnt5	-5.523	-2.045
ENSRNOG00000002800	Gdpd2	-5.855	-2.035
ENSRNOG00000011750*	Fam180a	-6.149	-2.022
