# Supplementary material for: Relcovaptan: a promising therapeutic agent in traumatic spinal cord injury that acts by modulating newly identified transcriptional regulators of aquaporins compared to tolvaptan
Source: Turk J Med Sci. 2025 Sep 22;55(6):1394–407. doi: 10.55730/1300-0144.6097 (PMC12779027; doi:10.55730/1300-0144.6097)
Supplement: Supplementary file 3 [file MedSci_55-6-1394_Supplementary-Data-3.pdf]

Supplementary Data 3: Transcription Factors.

Table S1. Differentially Expressed Transcription Factors. Transcription factors (TFs) are ordered from most upregulated to least downregulated. TFs that are differentially expressed in both treatments are marked with an asterisk.

Relcovaptan Treatment				Tolvaptan Treatment			
**Ensembl ID**	**Gene Symbol**	**Gene Name**	**log2(FC)**	**Ensembl ID**	**Gene Symbol**	**Gene Name**	**log2(FC)**
ENSRNOG00000012886	Maff *	MAF bZIP transcription factor F	3.364	ENSRNOG00000007491	Hoxb13	Homeobox B13	3.566
ENSRNOG00000012049	Sox7	SRY-box transcription factor 7	2.774	ENSRNOG00000012886	Maff *	MAF bZIP transcription factor F	2.899
ENSRNOG00000050869	Cebpd *	CCAAT/enhancer binding protein delta	2.641	ENSRNOG00000057347	Cebpb *	CCAAT/enhancer binding protein beta	2.303
ENSRNOG00000008015	Fos	Fos proto-oncogene, AP-1 transcription factor subunit	2.492	ENSRNOG00000050869	Cebpd *	CCAAT/enhancer binding protein delta	2.285
ENSRNOG00000005214	Plek	Pleckstrin	2.482	ENSRNOG00000022777	Six1 *	SIX homeobox 1	-2.911
ENSRNOG00000057347	Cebpb *	CCAAT/enhancer binding protein beta	2.262				
ENSRNOG00000024689	Hopx	HOP homeobox	2.150				
ENSRNOG00000003745	Atf3	Activating transcription factor 3	2.065				

ENSRNOG00000019660	Spib	Spi-B transcription factor	-2.489				
ENSRNOG00000004630	Rag1	Recombination activating 1	-3.055				
ENSRNOG00000022777	Six1 *	SIX homeobox 1	-3.812				

Table S2. Transcription Factors and Their Potential Binding Sites on AQP1.

TF	Source	Query	Start	Stop	Strand	Score	P-value	Q-value	Matched Sequence
**ATF3**	hTFtarget	ENA|BC090068|BC090068.1	244	254	+	12.6224	2.71e-05	128	GGTCACATCAG
**ATF3**	hTFtarget	ENA|BC090068|BC090068.1	244	254	+	11.6263	4.17e-05	192	GGTCACATCAG
**ATF3**	hTFtarget	ENA|BC090068|BC090068.1	721	732	+	9.39796	4.8e-05	128	TATGACTTCATC
**ATF3**	hTFtarget	ENA|BC090068|BC090068.1	721	732	-	8.53061	5.06e-05	0.14	GATGAAGTCATA
**ATF3**	hTFtarget	ENA|BC090068|BC090068.1	244	254	+	11.3131	5.08e-05	246	GGTCACATCAG
**ATF3**	hTFtarget	ENA|BC090068|BC090068.1	721	732	+	8.44898	5.33e-05	0.14	TATGACTTCATC
**ATF3**	hTFtarget	ENA|BC090068|BC090068.1	1750	1757	+	10.9355	6.59e-05	279	GTGACGTG
**ATF3**	hTFtarget	ENA|BC090068|BC090068.1	1086	1097	+	8.40816	6.88e-05	128	TTTGACATCATG
**ATF3**	hTFtarget	ENA|BC090068|BC090068.1	721	732	+	9.80808	7.17e-05	376	TATGACTTCATC
**ATF3**	hTFtarget	ENA|BC090068|BC090068.1	721	732	-	8.27551	7.32e-05	128	GATGAAGTCATA
**ATF3**	hTFtarget	ENA|BC090068|BC090068.1	1086	1097	+	8.27551	8.03e-05	174	TTTGACATCATG
**ATF3**	hTFtarget	ENA|BC090068|BC090068.1	1749	1759	+	10.3469	8.12e-05	138	TGTGACGTGTG
**ATF3**	hTFtarget	ENA|BC090068|BC090068.1	1389	1399	+	10.1735	8.79e-05	138	GATCACGTCTG
**ATF3**	hTFtarget	ENA|BC090068|BC090068.1	721	732	+	8.04082	8.89e-05	174	TATGACTTCATC
**ATF3**	hTFtarget	ENA|BC090068|BC090068.1	721	732	-	7.77551	9.94e-05	174	GATGAAGTCATA
**ATF3**	hTFtarget	ENA|BC090068|BC090068.1	244	254	+	12.6224	2.71e-05	128	GGTCACATCAG

**ATF3**	hTFtarget	ENA|BC090068|BC090068.1	244	254	+	11.6263	4.17e-05	192	GGTCACATCAG
**ATF3**	hTFtarget	ENA|BC090068|BC090068.1	721	732	+	9.39796	4.8e-05	128	TATGACTTCATC
**ATF3**	hTFtarget	ENA|BC090068|BC090068.1	721	732	-	8.53061	5.06e-05	0.14	GATGAAGTCATA
**ATF3**	hTFtarget	ENA|BC090068|BC090068.1	244	254	+	11.3131	5.08e-05	246	GGTCACATCAG
**ATF3**	hTFtarget	ENA|BC090068|BC090068.1	721	732	+	8.44898	5.33e-05	0.14	TATGACTTCATC
**ATF3**	hTFtarget	ENA|BC090068|BC090068.1	1750	1757	+	10.9355	6.59e-05	279	GTGACGTG
**ATF3**	hTFtarget	ENA|BC090068|BC090068.1	1086	1097	+	8.40816	6.88e-05	128	TTTGACATCATG
**ATF3**	hTFtarget	ENA|BC090068|BC090068.1	721	732	+	9.80808	7.17e-05	376	TATGACTTCATC
**ATF3**	hTFtarget	ENA|BC090068|BC090068.1	721	732	-	8.27551	7.32e-05	128	GATGAAGTCATA
**ATF3**	hTFtarget	ENA|BC090068|BC090068.1	1086	1097	+	8.27551	8.03e-05	174	TTTGACATCATG
**ATF3**	hTFtarget	ENA|BC090068|BC090068.1	1749	1759	+	10.3469	8.12e-05	138	TGTGACGTGTG
**ATF3**	hTFtarget	ENA|BC090068|BC090068.1	1389	1399	+	10.1735	8.79e-05	138	GATCACGTCTG
**ATF3**	hTFtarget	ENA|BC090068|BC090068.1	721	732	+	8.04082	8.89e-05	174	TATGACTTCATC
**ATF3**	hTFtarget	ENA|BC090068|BC090068.1	721	732	-	7.77551	9.94e-05	174	GATGAAGTCATA
**FOS**	hTFtarget	ENA|BC090068|BC090068.1	720	733	-	12.2069	1.45e-05	0.0763	GGATGAAGTCATAG
**FOS**	hTFtarget	ENA|BC090068|BC090068.1	720	733	+	10.1034	3.92e-05	103	CTATGACTTCATCC
**FOS**	hTFtarget	ENA|BC090068|BC090068.1	721	732	-	10.6162	4.8e-05	134	GATGAAGTCATA
**FOS**	hTFtarget	ENA|BC090068|BC090068.1	721	732	+	10.4747	5.18e-05	134	TATGACTTCATC

**FOS**	hTFtarget	ENA|BC090068|BC090068.1	720	733	-	12.2069	1.45e-05	0.0763	GGATGAAGTCATAG
**FOS**	hTFtarget	ENA|BC090068|BC090068.1	720	733	+	10.1034	3.92e-05	103	CTATGACTTCATCC
**FOS**	hTFtarget	ENA|BC090068|BC090068.1	721	732	-	10.6162	4.8e-05	134	GATGAAGTCATA
**FOS**	hTFtarget	ENA|BC090068|BC090068.1	721	732	+	10.4747	5.18e-05	134	TATGACTTCATC
**MAFF**	hTFtarget	ENA|BC090068|BC090068.1	1373	1387	-	10.8803	5.96e-05	245	CTGAGGCCTCAGCAT
**MAFF**	hTFtarget	ENA|BC090068|BC090068.1	1783	1797	-	9.37755	6.09e-05	317	ACATGACGCTGCAAT
**MAFF**	hTFtarget	ENA|BC090068|BC090068.1	809	824	-	9.9899	7.41e-05	359	TTGATATCATCAGCAT
**MAFF**	hTFtarget	ENA|BC090068|BC090068.1	1243	1258	+	8.9798	7.65e-05	377	TTCCTTAGGTAGGCAC
**MAFF**	hTFtarget	ENA|BC090068|BC090068.1	1373	1387	-	10.2636	7.97e-05	407	CTGAGGCCTCAGCAT
**MAFF**	hTFtarget	ENA|BC090068|BC090068.1	809	824	-	10.2394	8.07e-05	236	TTGATATCATCAGCAT
**MAFF**	hTFtarget	ENA|BC090068|BC090068.1	809	824	+	9.97887	9.43e-05	236	ATGCTGATGATATCAA
**MAFF**	hTFtarget	ENA|BC090068|BC090068.1	1373	1387	+	10.169	9.56e-05	245	ATGCTGAGGCCTCAG
**MAFF**	hTFtarget	ENA|BC090068|BC090068.1	1373	1387	-	10.8803	5.96e-05	245	CTGAGGCCTCAGCAT
**MAFF**	hTFtarget	ENA|BC090068|BC090068.1	1783	1797	-	9.37755	6.09e-05	317	ACATGACGCTGCAAT
**MAFF**	hTFtarget	ENA|BC090068|BC090068.1	809	824	-	9.9899	7.41e-05	359	TTGATATCATCAGCAT
**MAFF**	hTFtarget	ENA|BC090068|BC090068.1	1243	1258	+	8.9798	7.65e-05	377	TTCCTTAGGTAGGCAC
**MAFF**	hTFtarget	ENA|BC090068|BC090068.1	1373	1387	-	10.2636	7.97e-05	407	CTGAGGCCTCAGCAT
**MAFF**	hTFtarget	ENA|BC090068|BC090068.1	809	824	-	10.2394	8.07e-05	236	TTGATATCATCAGCAT

**MAFF**	hTFtarget	ENA|BC090068|BC090068.1	809	824	+	9.97887	9.43e-05	236	ATGCTGATGATATCAA
**MAFF**	hTFtarget	ENA|BC090068|BC090068.1	1373	1387	+	10.169	9.56e-05	245	ATGCTGAGGCCTCAG
**SOX7**	CISBP	ENA|BC090068|BC090068.1	1204	1220	-	-0.0714286	6.47e-05	339	TAACAATGGCAGAATCC

Table S3. Transcription Factors and Their Potential Binding Sites on AQP4.

TF	Source	Query	Start	Stop	Strand	Score	P-value	Q-value	Matched Sequence
**CEBPB**	hTFtarget	ENA|AF144082|AF144082.1	1768	1778	-	11.3556	7.32e-05	514	CATTTCATAAC
**CEBPB**	hTFtarget	ENA|AF144082|AF144082.1	1768	1778	-	11.3556	7.32e-05	514	CATTTCATAAC
**CEBPD**	hTFtarget	ENA|AF144082|AF144082.1	2741	2753	+	11.1193	6.78e-05	463	GTTTTCACAATAT
**CEBPD**	hTFtarget	ENA|AF144082|AF144082.1	2741	2753	+	11.1193	6.78e-05	463	GTTTTCACAATAT
**HOXB13**	hTFtarget	ENA|AF144082|AF144082.1	738	748	-	12	2.45e-05	172	ACTCGTAAAGT
**HOXB13**	hTFtarget	ENA|AF144082|AF144082.1	738	748	-	12.5152	2.6e-05	182	ACTCGTAAAGT
**HOXB13**	hTFtarget	ENA|AF144082|AF144082.1	2130	2139	-	11.8358	4.08e-05	283	CTTTATTGGC
**HOXB13**	hTFtarget	ENA|AF144082|AF144082.1	1161	1174	-	11.7619	4.21e-05	291	TTCCATTAAAATGT
**HOXB13**	hTFtarget	ENA|AF144082|AF144082.1	738	748	-	11.53	4.89e-05	343	ACTCGTAAAGT
**HOXB13**	hTFtarget	ENA|AF144082|AF144082.1	2130	2139	-	8.17308	8.31e-05	582	CTTTATTGGC
**HOXB13**	hTFtarget	ENA|AF144082|AF144082.1	1396	1406	-	7.82653	9.83e-05	345	TCTCCTAAAAC
**HOXB13**	hTFtarget	ENA|AF144082|AF144082.1	738	748	-	12	2.45e-05	172	ACTCGTAAAGT
**HOXB13**	hTFtarget	ENA|AF144082|AF144082.1	738	748	-	12.5152	2.6e-05	182	ACTCGTAAAGT
**HOXB13**	hTFtarget	ENA|AF144082|AF144082.1	2130	2139	-	11.8358	4.08e-05	283	CTTTATTGGC
**HOXB13**	hTFtarget	ENA|AF144082|AF144082.1	1161	1174	-	11.7619	4.21e-05	291	TTCCATTAAAATGT
**HOXB13**	hTFtarget	ENA|AF144082|AF144082.1	738	748	-	11.53	4.89e-05	343	ACTCGTAAAGT

**HOXB13**	hTFtarget	ENA|AF144082|AF144082.1	2130	2139	-	8.17308	8.31e-05	582	CTTTATTGGC
**HOXB13**	hTFtarget	ENA|AF144082|AF144082.1	1396	1406	-	7.82653	9.83e-05	345	TCTCCTAAAAC
**MAFF**	hTFtarget	ENA|AF144082|AF144082.1	2056	2071	+	12.2276	2.77e-05	192	TGGTCAGCATTGTGAG
**MAFF**	hTFtarget	ENA|AF144082|AF144082.1	1504	1519	-	10.5528	7.69e-05	0.22	CAGTCAGCTATATAAA
**MAFF**	hTFtarget	ENA|AF144082|AF144082.1	233	248	+	10.1789	9.51e-05	0.22	GACTCAGCATTGCCAC
**MAFF**	hTFtarget	ENA|AF144082|AF144082.1	2056	2071	+	12.2276	2.77e-05	192	TGGTCAGCATTGTGAG
**MAFF**	hTFtarget	ENA|AF144082|AF144082.1	1504	1519	-	10.5528	7.69e-05	0.22	CAGTCAGCTATATAAA
**MAFF**	hTFtarget	ENA|AF144082|AF144082.1	233	248	+	10.1789	9.51e-05	0.22	GACTCAGCATTGCCAC
**Six1**	CISBP	ENA|AF144082|AF144082.1	1258	1269	+	13.7523	1.06e-05	0.0744	GAAATCTGATCA
**SIX1**	JASPAR	ENA|AF144082|AF144082.1	1258	1268	+	12.6532	2.22e-05	155	GAAATCTGATC
**SIX1**	HOCOMOCO	ENA|AF144082|AF144082.1	1014	1027	-	12.2727	3.21e-05	162	GGAAATCTGAGGCC
**SIX1**	HOCOMOCO	ENA|AF144082|AF144082.1	1074	1087	-	11.7273	4.64e-05	162	TGAAACAAGAAACC
**Six1**	CISBP	ENA|AF144082|AF144082.1	1015	1026	-	12.0917	4.68e-05	164	GAAATCTGAGGC
**SOX7**	CISBP	ENA|AF144082|AF144082.1	2075	2090	-	7.03061	1.85e-05	128	ACCAATGGGTATTATT
**SOX7**	CISBP	ENA|AF144082|AF144082.1	1473	1488	-	4.32653	4.7e-05	128	AGCAATGGCAAGTGCT
**SOX7**	CISBP	ENA|AF144082|AF144082.1	2075	2090	+	3.73469	5.55e-05	128	AATAATACCCATTGGT
**SPIB**	hTFtarget	ENA|AF144082|AF144082.1	928	941	+	9.67347	4.62e-05	321	AAGAAGGGGAAGGA
**SPIB**	hTFtarget	ENA|AF144082|AF144082.1	929	941	+	11.4388	4.76e-05	0.33	AGAAGGGGAAGGA

**SPIB**	hTFtarget	ENA|AF144082|AF144082.1	2949	2964	+	10.5854	5.86e-05	318	GCTCACTTTCTCAGTC
**Spib**	HOCOMOCO	ENA|AF144082|AF144082.1	2987	3003	+	7.86364	6.36e-05	319	ATGGTAGGAACTGAAAA
**SPIB**	hTFtarget	ENA|AF144082|AF144082.1	2698	2704	-	11.2333	6.51e-05	455	AGAGGAA
**SPIB**	hTFtarget	ENA|AF144082|AF144082.1	2695	2706	-	10.7339	8.67e-05	601	AGAGAGGAAGCT
**SPIB**	hTFtarget	ENA|AF144082|AF144082.1	929	941	+	10.9592	8.79e-05	611	AGAAGGGGAAGGA
**SPIB**	hTFtarget	ENA|AF144082|AF144082.1	2986	3002	+	8.15152	8.88e-05	609	TATGGTAGGAACTGAAA
**Spib**	HOCOMOCO	ENA|AF144082|AF144082.1	1082	1098	-	6.75758	9.26e-05	319	GTAACAAGATGTGAAAC
**SPIB**	hTFtarget	ENA|AF144082|AF144082.1	2987	3002	-	9.69919	9.31e-05	318	TTTCAGTTCCTACCAT
**SPIB**	hTFtarget	ENA|AF144082|AF144082.1	928	941	+	9.67347	4.62e-05	321	AAGAAGGGGAAGGA
**SPIB**	hTFtarget	ENA|AF144082|AF144082.1	929	941	+	11.4388	4.76e-05	0.33	AGAAGGGGAAGGA
**SPIB**	hTFtarget	ENA|AF144082|AF144082.1	2949	2964	+	10.5854	5.86e-05	318	GCTCACTTTCTCAGTC
**SPIB**	hTFtarget	ENA|AF144082|AF144082.1	2698	2704	-	11.2333	6.51e-05	455	AGAGGAA
**SPIB**	hTFtarget	ENA|AF144082|AF144082.1	2695	2706	-	10.7339	8.67e-05	601	AGAGAGGAAGCT
**SPIB**	hTFtarget	ENA|AF144082|AF144082.1	929	941	+	10.9592	8.79e-05	611	AGAAGGGGAAGGA
**SPIB**	hTFtarget	ENA|AF144082|AF144082.1	2986	3002	+	8.15152	8.88e-05	609	TATGGTAGGAACTGAAA
**SPIB**	hTFtarget	ENA|AF144082|AF144082.1	2987	3002	-	9.69919	9.31e-05	318	TTTCAGTTCCTACCAT

Table S4. Transcription Factors and Their Potential Binding Sites on AQP9.

TF	Source	Query	Start	Stop	Strand	Score	P-value	Q-value	Matched Sequence
**ATF3**	hTFtarget	ENA|AF016406|AF016406.1	1179	1190	-	8.60204	6.39e-05	0.19	GGTGACATCTTC
**Atf3**	CISBP	ENA|AF016406|AF016406.1	1117	1125	-	8.80198	8.19e-05	122	TGATGACAC
**Atf3**	CISBP	ENA|AF016406|AF016406.1	1442	1450	-	8.80198	8.19e-05	122	TGATGACAC
**ATF3**	hTFtarget	ENA|AF016406|AF016406.1	1185	1192	+	10.7258	8.78e-05	241	GTCACCTG
**ATF3**	hTFtarget	ENA|AF016406|AF016406.1	1179	1190	-	8.60204	6.39e-05	0.19	GGTGACATCTTC
**ATF3**	hTFtarget	ENA|AF016406|AF016406.1	1185	1192	+	10.7258	8.78e-05	241	GTCACCTG
**CEBPB**	hTFtarget	ENA|AF016406|AF016406.1	371	382	-	12.2576	5.12e-05	118	GCTTGGGCAATA
**Cebpb**	CISBP	ENA|AF016406|AF016406.1	372	381	-	11.4018	5.97e-05	177	CTTGGGCAAT
**Cebpb**	HOCOMOCO	ENA|AF016406|AF016406.1	372	382	-	11.8939	6.11e-05	114	GCTTGGGCAAT
**CEBPB**	hTFtarget	ENA|AF016406|AF016406.1	502	515	+	11.7449	6.35e-05	138	TGTCTTTTGCAATG
**Cebpb**	HOCOMOCO	ENA|AF016406|AF016406.1	504	514	+	11.6212	7.7e-05	114	TCTTTTGCAAT

**CEBPB**	hTFtarget	ENA|AF016406|AF016406.1	504	515	+	11.5909	7.94e-05	118	TCTTTTGCAATG
**CEBPB**	hTFtarget	ENA|AF016406|AF016406.1	371	384	-	11.0714	9.35e-05	138	CCGCTTGGGCAATA
**CEBPB**	hTFtarget	ENA|AF016406|AF016406.1	371	382	-	12.2576	5.12e-05	118	GCTTGGGCAATA
**CEBPB**	hTFtarget	ENA|AF016406|AF016406.1	502	515	+	11.7449	6.35e-05	138	TGTCTTTTGCAATG
**CEBPB**	hTFtarget	ENA|AF016406|AF016406.1	504	515	+	11.5909	7.94e-05	118	TCTTTTGCAATG
**CEBPB**	hTFtarget	ENA|AF016406|AF016406.1	371	384	-	11.0714	9.35e-05	138	CCGCTTGGGCAATA
**Cebpd**	HOCOMOCO	ENA|AF016406|AF016406.1	372	382	-	11.9398	2.25e-05	0.0668	GCTTGGGCAAT
**CEBPD**	hTFtarget	ENA|AF016406|AF016406.1	372	382	+	12.0182	2.89e-05	0.0857	ATTGCCCAAGC
**CEBPD**	hTFtarget	ENA|AF016406|AF016406.1	371	383	+	11.1009	6.84e-05	0.0925	TATTGCCCAAGCG
**CEBPD**	hTFtarget	ENA|AF016406|AF016406.1	1459	1471	+	10.8991	7.64e-05	0.0925	GTTTTCACAATCT
**CEBPD**	hTFtarget	ENA|AF016406|AF016406.1	503	515	-	10.5413	9.35e-05	0.0925	CATTGCAAAAGAC
**CEBPD**	hTFtarget	ENA|AF016406|AF016406.1	372	382	+	12.0182	2.89e-05	0.0857	ATTGCCCAAGC

**CEBPD**	hTFtarget	ENA|AF016406|AF016406.1	371	383	+	11.1009	6.84e-05	0.0925	TATTGCCCAAGCG
**CEBPD**	hTFtarget	ENA|AF016406|AF016406.1	1459	1471	+	10.8991	7.64e-05	0.0925	GTTTTCACAATCT
**CEBPD**	hTFtarget	ENA|AF016406|AF016406.1	503	515	-	10.5413	9.35e-05	0.0925	CATTGCAAAAGAC
**FOS**	hTFtarget	ENA|AF016406|AF016406.1	1179	1190	-	11.9184	3.43e-05	102	GGTGACATCTTC
**FOS**	hTFtarget	ENA|AF016406|AF016406.1	1179	1190	-	11.9184	3.43e-05	102	GGTGACATCTTC
**Maff**	HOCOMOCO	ENA|AF016406|AF016406.1	401	422	-	9.25253	8.53e-05	0.25	GATAGTGATGATCCCGCCAAAA
**Six1**	CISBP	ENA|AF016406|AF016406.1	1380	1391	+	13.5138	1.38e-05	41	GAAACTTGACCA
**SIX1**	HOCOMOCO	ENA|AF016406|AF016406.1	1379	1392	+	12.7273	2.32e-05	0.0686	TGAAACTTGACCAT
**SIX1**	JASPAR	ENA|AF016406|AF016406.1	1380	1390	+	11.6129	4.53e-05	0.0952	GAAACTTGACC
**SIX1**	HOCOMOCO	ENA|AF016406|AF016406.1	1102	1115	+	11.6667	4.83e-05	0.0715	AGAAACACGAGCTC
**SIX1**	JASPAR	ENA|AF016406|AF016406.1	123	133	-	11.0887	6.41e-05	0.0952	AAAACCTGATT
**Six1**	CISBP	ENA|AF016406|AF016406.1	1103	1114	+	10.9174	8.64e-05	128	GAAACACGAGCT

**SIX1**	HOCOMOCO	ENA|AF016406|AF016406.1	1452	1465	-	10.5152	9.76e-05	0.0963	TGAAAACAGAGCCT
**SOX7**	CISBP	ENA|AF016406|AF016406.1	671	686	+	3.70408	5.6e-05	166	AACAGCATTCATTTTT
**Spib**	HOCOMOCO	ENA|AF016406|AF016406.1	1159	1175	-	9.63636	3.32e-05	0.0956	GAAGAAAGAACTGGATG
**SPIB**	hTFtarget	ENA|AF016406|AF016406.1	1160	1176	-	9.84848	4.54e-05	131	TGAAGAAAGAACTGGAT
**SPIB**	hTFtarget	ENA|AF016406|AF016406.1	543	556	-	8	9.15e-05	0.27	TAAAATGGGAACTT
**SPIB**	hTFtarget	ENA|AF016406|AF016406.1	1160	1176	-	9.84848	4.54e-05	131	TGAAGAAAGAACTGGAT
**SPIB**	hTFtarget	ENA|AF016406|AF016406.1	543	556	-	8	9.15e-05	0.27	TAAAATGGGAACTT

Table S5. Transcription Factors and Their Potential Binding Sites on AQP11.

TF	Source	Query	Start	Stop	Strand	Score	P-value	Q-value	Matched Sequence
**ATF3**	hTFtarget	ENA|AB023644|AB023644.1	320	330	-	11.4747	5.48e-05	125	GATCACGCGGG
**Atf3**	HOCOMOCO	ENA|AB023644|AB023644.1	1024	1032	+	10.8182	8.55e-05	221	CTGAGTAAC
**ATF3**	hTFtarget	ENA|AB023644|AB023644.1	320	330	-	11.4747	5.48e-05	125	GATCACGCGGG
**CEBPD**	hTFtarget	ENA|AB023644|AB023644.1	1008	1018	+	10.6818	9.05e-05	226	GCTGCACAACA
**CEBPD**	hTFtarget	ENA|AB023644|AB023644.1	1008	1018	+	10.6818	9.05e-05	226	GCTGCACAACA
**Maff**	HOCOMOCO	ENA|AB023644|AB023644.1	420	441	+	14.4747	4.43e-06	0.0112	CCAACTGCTGAGCGAGCAGGAC
**MAFF**	hTFtarget	ENA|AB023644|AB023644.1	420	437	-	12816	1.47e-05	0.0246	TGCTCGCTCAGCAGTTGG
**MAFF**	hTFtarget	ENA|AB023644|AB023644.1	425	442	+	12424	1.96e-05	0.0246	TGCTGAGCGAGCAGGACT
**MAFF**	hTFtarget	ENA|AB023644|AB023644.1	419	436	-	12.2667	3.05e-05	0.0774	GCTCGCTCAGCAGTTGGA
**Maff**	HOCOMOCO	ENA|AB023644|AB023644.1	586	607	-	10899	3.6e-05	0.0416	TAACCAGCTGAGCCATCAACCT
**MAFF**	hTFtarget	ENA|AB023644|AB023644.1	425	439	+	10.5408	3.62e-05	0.0926	TGCTGAGCGAGCAGG
**MAFF**	hTFtarget	ENA|AB023644|AB023644.1	424	438	-	11.2746	4.57e-05	0.0707	CTGCTCGCTCAGCAG
**MAFF**	hTFtarget	ENA|AB023644|AB023644.1	424	438	+	11	0,00005	0.0647	CTGCTGAGCGAGCAG
**MAFF**	hTFtarget	ENA|AB023644|AB023644.1	424	438	-	10.9364	5.21e-05	0.0647	CTGCTCGCTCAGCAG
**MAFF**	hTFtarget	ENA|AB023644|AB023644.1	424	438	+	10.9507	5.69e-05	0.0707	CTGCTGAGCGAGCAG
**MAFF**	hTFtarget	ENA|AB023644|AB023644.1	586	606	-	10.16	5.85e-05	0.0784	AACCAGCTGAGCCATCAACCT

**MAFF**	hTFtarget	ENA|AB023644|AB023644.1	421	441	+	10072	6.13e-05	0.0784	CAACTGCTGAGCGAGCAGGAC
**Maff**	HOCOMOCO	ENA|AB023644|AB023644.1	585	606	+	9.85859	6.25e-05	0.0416	GAGGTTGATGGCTCAGCTGGTT
**Maff**	HOCOMOCO	ENA|AB023644|AB023644.1	421	442	-	9.76768	6.55e-05	0.0416	AGTCCTGCTCGCTCAGCAGTTG
**MAFF**	hTFtarget	ENA|AB023644|AB023644.1	585	602	-	10504	7.53e-05	0.0629	AGCTGAGCCATCAACCTC
**MAFF**	hTFtarget	ENA|AB023644|AB023644.1	424	438	-	9.11224	8.11e-05	205	CTGCTCGCTCAGCAG
**MAFF**	hTFtarget	ENA|AB023644|AB023644.1	588	602	-	8.61224	8.45e-05	108	AGCTGAGCCATCAAC
**MAFF**	hTFtarget	ENA|AB023644|AB023644.1	421	441	-	9224	9.66e-05	0.0823	GTCCTGCTCGCTCAGCAGTTG
**MAFF**	hTFtarget	ENA|AB023644|AB023644.1	420	437	-	12816	1.47e-05	0.0246	TGCTCGCTCAGCAGTTGG
**MAFF**	hTFtarget	ENA|AB023644|AB023644.1	425	442	+	12424	1.96e-05	0.0246	TGCTGAGCGAGCAGGACT
**MAFF**	hTFtarget	ENA|AB023644|AB023644.1	419	436	-	12.2667	3.05e-05	0.0774	GCTCGCTCAGCAGTTGGA
**MAFF**	hTFtarget	ENA|AB023644|AB023644.1	425	439	+	10.5408	3.62e-05	0.0926	TGCTGAGCGAGCAGG
**MAFF**	hTFtarget	ENA|AB023644|AB023644.1	424	438	-	11.2746	4.57e-05	0.0707	CTGCTCGCTCAGCAG
**MAFF**	hTFtarget	ENA|AB023644|AB023644.1	424	438	+	11	0,00005	0.0647	CTGCTGAGCGAGCAG
**MAFF**	hTFtarget	ENA|AB023644|AB023644.1	424	438	-	10.9364	5.21e-05	0.0647	CTGCTCGCTCAGCAG
**MAFF**	hTFtarget	ENA|AB023644|AB023644.1	424	438	+	10.9507	5.69e-05	0.0707	CTGCTGAGCGAGCAG
**MAFF**	hTFtarget	ENA|AB023644|AB023644.1	586	606	-	10.16	5.85e-05	0.0784	AACCAGCTGAGCCATCAACCT
**MAFF**	hTFtarget	ENA|AB023644|AB023644.1	421	441	+	10072	6.13e-05	0.0784	CAACTGCTGAGCGAGCAGGAC
**MAFF**	hTFtarget	ENA|AB023644|AB023644.1	585	602	-	10504	7.53e-05	0.0629	AGCTGAGCCATCAACCTC

**MAFF**	hTFtarget	ENA|AB023644|AB023644.1	424	438	-	9.11224	8.11e-05	205	CTGCTCGCTCAGCAG
**MAFF**	hTFtarget	ENA|AB023644|AB023644.1	588	602	-	8.61224	8.45e-05	108	AGCTGAGCCATCAAC
**MAFF**	hTFtarget	ENA|AB023644|AB023644.1	421	441	-	9224	9.66e-05	0.0823	GTCCTGCTCGCTCAGCAGTTG
**Spib**	HOCOMOCO	ENA|AB023644|AB023644.1	894	910	-	9.18182	3.94e-05	0.0979	AGCACGGGAAGTGCAGC
**SPIB**	hTFtarget	ENA|AB023644|AB023644.1	895	911	-	9.9697	4.32e-05	107	AAGCACGGGAAGTGCAG
**SPIB**	hTFtarget	ENA|AB023644|AB023644.1	895	910	+	11.1057	4.42e-05	0.11	CTGCACTTCCCGTGCT
**SPIB**	hTFtarget	ENA|AB023644|AB023644.1	898	909	-	10.9184	8.76e-05	215	GCACGGGAAGTG
**SPIB**	hTFtarget	ENA|AB023644|AB023644.1	898	909	-	9.87755	8.89e-05	226	GCACGGGAAGTG
**SPIB**	hTFtarget	ENA|AB023644|AB023644.1	895	911	-	9.9697	4.32e-05	107	AAGCACGGGAAGTGCAG
**SPIB**	hTFtarget	ENA|AB023644|AB023644.1	895	910	+	11.1057	4.42e-05	0.11	CTGCACTTCCCGTGCT
**SPIB**	hTFtarget	ENA|AB023644|AB023644.1	898	909	-	10.9184	8.76e-05	215	GCACGGGAAGTG
**SPIB**	hTFtarget	ENA|AB023644|AB023644.1	898	909	-	9.87755	8.89e-05	226	GCACGGGAAGTG

Table S6. Transcription Factor Binding Sites and Expression Patterns. Transcription factors (TFs) that are differentially expressed after Relcovaptan and Tolvaptan treatment in traumatic spinal cord injury and their potential binding sites with q < 1.0 on differentially expressed aquaporin (AQP) genes.

Relcovaptan Treatment					TF	Tolvaptan Treatment				
**Expression of TF**	**AQP1 (↓)**	**AQP4 (↓)**	**AQP9 (–)**	**AQP11 (↓)**		**Expression of TF**	**AQP1 (↑)**	**AQP4 (↑)**	**AQP9 (↑)**	**AQP11 (↑)**
↑	**X**		**X**		**ATF3**	–				
↑			X		**CEBPD**	↑			X	
↑	**X**				**FOS**	–				
↑		X	X	X	**MAFF**	↑		X	X	X
↓		X	X		**SIX1**	↓		X	X	
↓		**X**	**X**	**X**	**SPIB**	–				

↑: Upregulated
–: Not differentially expressed
↓: Downregulated
X: Presence of a TF binding site (TFs specific to relcovaptan treatment are indicated by a blue bold X)
