# Supplementary material for: Relcovaptan: a promising therapeutic agent in traumatic spinal cord injury that acts by modulating newly identified transcriptional regulators of aquaporins compared to tolvaptan
Source: Turk J Med Sci. 2025 Sep 22;55(6):1394–407. doi: 10.55730/1300-0144.6097 (PMC12779027; doi:10.55730/1300-0144.6097)
Supplement: Supplementary file 4 [file MedSci_55-6-1394_Supplementary-Data-4.pdf]

Supplementary Data 4: GO Analysis Results.

Table S1. GO Biological Process Analysis Results of Relcovaptan Treatment. Top 10 GO terms revealed by GO analysis of biological processes; analysis performed with upregulated genes after Relcovaptan treatment.

Term	Overlap	P-Value	Adjusted P-Value	Genes
cytokine-mediated signaling pathway	31/633	1.05E-20	5.37E-17	IFITM3; IL1RN; IFITM1; CXCL9; CDKN1A; CEBPD; CSF2RB; CXCL1; CXCL3; CXCL2; IFIT3; SOCS3; MT2A; CCL7; CCL3; HMOX1; CCL2; TIMP1; JUNB; TNFRSF12A; IL1R2; LIF; OSM; FOS; OSMR; TNFRSF1B; IL1B; CLCF1; LCN2; SDC1; BIRC3
cellular response to cytokine stimulus	25/456	7.08E-18	1.81E-14	IL1RN; CDKN1A; CEBPD; CSF2RB; CXCL1; CXCL2; SOCS3; MT2A; CCL7; CCL3; HMOX1; CCL2; TIMP1; JUNB; IL1R2; LIF; OSM; ACOD1; FOS; OSMR; TNFRSF1B; IL1B; CLCF1; LCN2; SDC1
inflammatory response	16/252	1.08E-12	1.83E-9	CXCL9; CEBPB; TNFAIP6; CXCL1; FOS; CXCL3; TNFRSF1B; CXCL2; THBS1; CCL7; IL1B; BDKRB2; CCL3; CCL2; PTX3; FCGR2B
response to molecule of bacterial origin	11/98	9.33E-12	1.19E-8	CXCL9; SLPI; ACOD1; CXCL1; CD14; TRIB1; CXCL3; TNFRSF1B; FCGR2B; JUNB; CXCL2
regulation of cell proliferation	23/740	2.53E-11	2.59E-8	IFITM1; CXCL9; CDKN1A; IGFBP3; LIF; OSM; CXCL1; CXCL3; OSMR; TNFRSF1B; CXCL2; THBS1; IFIT3; ESM1; GPNMB; ADAMTS1; IL1B; CLCF1; PDPN; TIMP1; SGK1; JUNB; SOX7
response to lipopolysaccharide	12/155	8.55E-11	7.27E-8	CXCL9; SLPI; SERPINE1; ACOD1; CCL2; CXCL1; CD14; TRIB1; CXCL3; TNFRSF1B; JUNB; CXCL2
positive regulation of leukocyte chemotaxis	8/61	1.99E-9	1.45E-6	CXCL9; CCL7; SERPINE1; CCL3; CXCL1; CXCL3; CXCL2; THBS1
chemokine-mediated signaling pathway	7/52	1.76E-8	1.12E-5	CXCL9; CCL7; CCL3; CCL2; CXCL1; CXCL3; CXCL2
negative regulation of cell proliferation	13/363	1.56E-7	8.82E-5	IFITM1; CDKN1A; IGFBP3; OSM; CXCL1; IFIT3; GPNMB; ADAMTS1; IL1B; PDPN; HMOX1; TRIB1; SOX7
positive regulation of chemotaxis	6/48	3.03E-7	1.55E-4	CXCL9; HSPB1; CXCL1; CXCL3; CXCL2; THBS1

Table S2. GO Biological Process Analysis Results of Relcovaptan Treatment. Top 10 GO terms revealed by GO analysis of biological processes; analysis performed with downregulated genes after Relcovaptan treatment.

Term	Overlap	P-Value	Adjusted P-Value	Genes
muscle contraction	20/137	5.33E-26	2.72E-22	RYR1; MYLK2; MYBPC1; MYBPC2; ACTN3; ACTN2; TNNC2; NEB; MYOM2; LMOD3; KLHL41; TRDN; ACTA1; MYH2; MYL1; TNNT3; HRC; TNNI2; SCN4A; MYH6
muscle filament sliding	12/38	2.42E-20	6.17E-17	ACTA1; MYBPC1; MYH2; MYBPC2; ACTN3; ACTN2; MYL1; TNNC2; TNNT3; TNNI2; NEB; MYH6
actin-myosin filament sliding	12/38	2.42E-20	4.11E-17	ACTA1; MYBPC1; MYH2; MYBPC2; ACTN3; ACTN2; MYL1; TNNC2; TNNT3; TNNI2; NEB; MYH6
myofibril assembly	12/47	4.53E-19	5.78E-16	MYLK2; MYBPC1; MYBPC2; OBSCN; ACTN2; TNNT3; CASQ1; MYOZ1; MYOM2; LMOD3; KLHL41; MYH6
actomyosin structure organization	12/71	1.03E-16	1.05E-13	MYLK2; MYBPC1; MYBPC2; OBSCN; ACTN2; TNNT3; CASQ1; MYOZ1; LMOD3; MYOM2; KLHL41; MYH6
sarcomere organization	9/31	3.90E-15	3.32E-12	MYBPC1; MYBPC2; OBSCN; ACTN2; TNNT3; CASQ1; MYOM2; KLHL41; MYH6
striated muscle contraction	10/61	5.79E-14	4.22E-11	MYLK2; MYBPC1; MYBPC2; TNNC2; TNNT3; TNNI2; LMOD3; MYOM2; KLHL41; MYH6
muscle fiber development	6/27	1.09E-9	6.95E-7	RYR1; ACTA1; MYOM2; LMOD3; KLHL41; MYH6
skeletal muscle tissue development	6/39	1.15E-8	6.55E-6	RYR1; MYLK2; ACTA1; SIX1; LMOD3; KLHL41
skeletal muscle fiber development	4/12	1.27E-7	6.46E-5	RYR1; ACTA1; KLHL41; LMOD3

Table S3. GO Biological Process Analysis Results of Tolvaptan Treatment. Top 10 GO terms revealed by GO analysis of biological processes; analysis performed with upregulated genes after Tolvaptan treatment.

Term	Overlap	P-Value	Adjusted P-Value	Genes
cytokine-mediated signaling pathway	24/633	6.77E-14	3.45E-10	IL11; CSF3; IL1RN; IFITM1; CDKN1A; CEBPD; IL1R2; LIF; OSM; CSF2RB; CXCL1; CXCL3; CXCL2; IFIT3; NFKBIA; SOCS3; MT2A; IL1B; CLCF1; CCL3; SDC1; CCL2; LTB; JUNB
cellular response to cytokine stimulus	20/456	7.56E-13	1.93E-9	IL11; CSF3; IL1RN; CDKN1A; CEBPD; IL1R2; LIF; OSM; ACOD1; CSF2RB; CXCL1; CXCL2; SOCS3; MT2A; IL1B; CLCF1; CCL3; SDC1; CCL2; JUNB
response to molecule of bacterial origin	9/98	3.75E-9	6.37E-6	SLPI; NOS3; ACOD1; CXCL1; CD14; CXCL3; FCGR2B; JUNB; CXCL2
regulation of cell proliferation	20/740	4.05E-9	5.16E-6	IL11; CSF3; IFITM1; CDKN1A; NOS3; LIF; OSM; CXCL1; CXCL3; CXCL2; THBS1; IFIT3; FGR; ESM1; GPNMB; IL1B; CLCF1; CXCR2; SGK1; JUNB
response to lipopolysaccharide	10/155	1.54E-8	1.57E-5	SLPI; NOS3; SERPINE1; ACOD1; CCL2; CXCL1; CD14; CXCL3; JUNB; CXCL2
inflammatory response	12/252	1.61E-8	1.37E-5	CEBPB; IL1B; CXCR2; BDKRB2; CCL3; CCL2; ADAM8; CXCL1; CXCL3; FCGR2B; CXCL2; THBS1
positive regulation of leukocyte migration	6/34	3.02E-8	2.20E-5	LGALS3; F2RL1; CXCL1; ADAM8; CXCL3; CXCL2
positive regulation of leukocyte chemotaxis	7/61	4.57E-8	2.91E-5	SERPINE1; CCL3; F2RL1; CXCL1; CXCL3; CXCL2; THBS1
negative regulation of hormone secretion	4/13	6.18E-7	3.50E-4	IL11; IL1B; LIF; OSM
positive regulation of chemotaxis	5/48	6.81E-6	0.00347	F2RL1; CXCL1; CXCL3; CXCL2; THBS1

Table S4. GO Biological Process Analysis Results of Tolvaptan Treatment. Top 10 GO terms revealed by GO analysis of biological processes; analysis performed with downregulated genes after Tolvaptan treatment.

Term	Overlap	P-Value	Adjusted P-Value	Genes
muscle contraction	24/137	1.23E-34	6.26E-31	RYR1; MYOM1; MYLK2; MYBPC1; MYBPC2; ACTN3; ACTN2; TPM2; PGAM2; TNNC2; NEB; KLHL41; TRDN; MYLPF; ACTA1; MYH2; DES; MYL1; TNNT3; HRC; TNNI2; SCN4A; MYH6; MYH7
muscle filament sliding	15/38	8.46E-28	2.16E-24	MYBPC1; MYBPC2; ACTN3; ACTN2; TPM2; TNNC2; NEB; ACTA1; MYH2; DES; MYL1; TNNT3; TNNI2; MYH6; MYH7
actin-myosin filament sliding	15/38	8.46E-28	1.44E-24	MYBPC1; MYBPC2; ACTN3; ACTN2; TPM2; TNNC2; NEB; ACTA1; MYH2; DES; MYL1; TNNT3; TNNI2; MYH6; MYH7
myofibril assembly	12/47	1.02E-19	1.30E-16	MYLK2; MYOM1; MYBPC1; MYBPC2; OBSCN; ACTN2; TNNT3; CASQ1; MYOZ1; MYPN; KLHL41; MYH6
striated muscle contraction	12/61	3.27E-18	3.34E-15	MYLK2; MYOM1; MYBPC1; MYBPC2; JSRP1; TNNC2; PGAM2; TNNT3; TNNI2; KLHL41; MYH6; MYH7
sarcomere organization	10/31	9.21E-18	7.83E-15	MYOM1; MYBPC1; MYBPC2; OBSCN; ACTN2; TNNT3; CASQ1; MYPN; KLHL41; MYH6
actomyosin structure organization	12/71	2.34E-17	1.70E-14	MYLK2; MYOM1; MYBPC1; MYBPC2; OBSCN; ACTN2; TNNT3; CASQ1; MYOZ1; MYPN; KLHL41; MYH6
muscle fiber development	6/27	5.34E-10	3.41E-7	RYR1; MYOM1; ACTA1; MYPN; KLHL41; MYH6
skeletal muscle contraction	5/18	4.65E-9	2.64E-6	JSRP1; TNNC2; TNNT3; TNNI2; MYH7
skeletal muscle tissue development	6/39	5.69E-9	2.90E-6	RYR1; MYLPF; MYLK2; ACTA1; SIX1; KLHL41

Table S5. GO Molecular Function Analysis Results of Relcovaptan Treatment. Top 10 GO terms revealed by GO analysis of molecular functions; analysis performed with upregulated genes after Relcovaptan treatment.

Term	Overlap	P-Value	Adjusted P-Value	Genes
cytokine activity	13/155	4.83E-12	5.56E-9	IL1RN; CXCL9; LIF; OSM; CXCL1; CXCL3; CXCL2; CCL7; IL1B; CLCF1; CCL3; CCL2; TIMP1
chemokine activity	7/46	7.23E-9	4.16E-6	CXCL9; CCL7; CCL3; CCL2; CXCL1; CXCL3; CXCL2
chemokine receptor binding	7/49	1.14E-8	4.39E-6	CXCL9; CCL7; CCL3; CCL2; CXCL1; CXCL3; CXCL2
CXCR chemokine receptor binding	4/17	2.25E-6	6.47E-4	CXCL9; CXCL1; CXCL3; CXCL2
protein heterodimerization activity	7/265	8.17E-4	0.188	CEBPB; BCL2A1; CLCF1; BDKRB2; FOS; ATF3; TLR2
cytokine receptor binding	5/137	0.00113	0.217	IL1RN; IL1B; CLCF1; LIF; OSM
CCR chemokine receptor binding	3/38	0.00132	0.216	CCL7; CCL3; CCL2
immunoglobulin binding	2/11	0.00171	0.246	LGALS3; FCGR2B
galanin receptor activity	2/11	0.00171	0.219	GAL; GPR84
neuropeptide receptor binding	2/11	0.00171	0.197	GAL; UCN2

Table S6. GO Molecular Function Analysis Results of Relcovaptan Treatment. Top 10 GO terms revealed by GO analysis of molecular functions; analysis performed with downregulated genes after Relcovaptan treatment.

Term	Overlap	P-Value	Adjusted P-Value	Genes
actin binding	11/254	7.06E-9	8.12E-6	GAS2L3; MYBPC1; MYBPC2; TNNC2; TNNT3; NRAP; TNNI2; XIRP2; MYOZ1; MYOM2; LMOD3
titin binding	4/14	2.55E-7	1.47E-4	MYBPC1; OBSCN; ACTN2; ANKRD23
muscle alpha-actinin binding	4/16	4.60E-7	1.76E-4	MYBPC1; MYBPC2; NRAP; MYOM2
alpha-actinin binding	4/29	5.76E-6	0.00166	MYBPC1; MYBPC2; NRAP; MYOM2
inorganic anion transmembrane transporter activity	4/48	4.45E-5	0.0102	SLC22A6; SLC26A7; SLC4A1; SLC5A5
ion antiporter activity	3/18	5.18E-5	0.00995	SLC22A6; SLC26A7; SLC4A1
anion:anion antiporter activity	3/21	8.38E-5	0.0138	SLC22A6; SLC26A7; SLC4A1
integrin binding	4/94	6.05E-4	0.0870	IBSP; ACTN3; ACTN2; WISP2
calcium ion binding	6/284	0.00110	0.141	RYR1; MMP13; TNNC2; TNNT3; HRC; CASQ1
tropomyosin binding	2/14	0.00146	0.168	TNNT3; LMOD3

Table S7. GO Molecular Function Analysis Results of Tolvaptan Treatment. Top 10 GO terms revealed by GO analysis of molecular functions; analysis performed with upregulated genes after Tolvaptan treatment.

Term	Overlap	P-Value	Adjusted P-Value	Genes
cytokine activity	12/155	6.23E-11	7.17E-8	IL11; CSF3; IL1RN; IL1B; CLCF1; LIF; CCL3; OSM; CCL2; CXCL1; CXCL3; CXCL2
chemokine activity	5/46	5.50E-6	0.00316	CCL3; CCL2; CXCL1; CXCL3; CXCL2
chemokine receptor binding	5/49	7.55E-6	0.00290	CCL3; CCL2; CXCL1; CXCL3; CXCL2
cytokine receptor binding	7/137	1.14E-5	0.00328	IL11; CSF3; IL1RN; IL1B; CLCF1; LIF; OSM
growth factor activity	5/69	4.07E-5	0.00938	IL11; CSF3; CLCF1; LIF; OSM
CXCR chemokine receptor binding	3/17	1.07E-4	0.0205	CXCL1; CXCL3; CXCL2
transforming growth factor beta binding	3/19	1.51E-4	0.0248	CD109; LTBP2; THBS1
immunoglobulin binding	2/11	0.00162	0.234	LGALS3; FCGR2B
growth factor receptor binding	4/92	0.00173	0.221	IL11; CSF3; ESM1; IL1B
integrin binding	4/94	0.00187	0.215	ESM1; GPNMB; IL1B; THBS1

Table S8. GO Molecular Function Analysis Results of Tolvaptan Treatment. Top 10 GO terms revealed by GO analysis of molecular functions; analysis performed with downregulated genes after Tolvaptan treatment.

Term	Overlap	P-Value	Adjusted P-Value	Genes
actin binding	12/254	1.26E-10	1.45E-7	MYOM1; MYBPC1; MYBPC2; TPM2; TNNC2; TNNT3; NRAP; TNNI2; XIRP2; MYOZ1; ABRA; MYPN
muscle alpha-actinin binding	5/16	2.39E-9	1.37E-6	MYOM1; MYBPC1; MYBPC2; NRAP; MYPN
alpha-actinin binding	5/29	6.25E-8	2.40E-5	MYOM1; MYBPC1; MYBPC2; NRAP; MYPN
titin binding	4/14	1.59E-7	4.58E-5	MYBPC1; OBSCN; ACTN2; ANKRD23
actin filament binding	6/127	7.00E-6	0.00161	MYOM1; MYBPC1; MYBPC2; TPM2; TNNC2; MYPN
ion antiporter activity	3/18	3.66E-5	0.00702	SLC22A6; SLC26A7; SLC22A8
microfilament motor activity	3/18	3.66E-5	0.00602	MYH2; MYH6; MYH7
anion:anion antiporter activity	3/21	5.92E-5	0.00851	SLC22A6; SLC26A7; SLC22A8
actin-dependent ATPase activity	2/9	4.65E-4	0.0595	MYH6; MYH7
calcium ion binding	6/284	5.97E-4	0.0687	RYR1; MB; TNNC2; TNNT3; HRC; CASQ1
