# Supplementary material for: Relcovaptan: a promising therapeutic agent in traumatic spinal cord injury that acts by modulating newly identified transcriptional regulators of aquaporins compared to tolvaptan
Source: Turk J Med Sci. 2025 Sep 22;55(6):1394–407. doi: 10.55730/1300-0144.6097 (PMC12779027; doi:10.55730/1300-0144.6097)
Supplement: Supplementary file 5 [file MedSci_55-6-1394_Supplementary-Data-5.pdf]

Supplementary Data 5: Pathway Analysis Results.

Table S1. Pathway Analysis Results of Relcovaptan Treatment. The 10 most significant pathways obtained from WikiPathways analysis of differentially expressed genes after Relcovaptan treatment.

Term	Overlap	P-Value	Adjusted P-Value	Genes
Striated Muscle Contraction	12/45	1.21E-14	2.13E-12	ACTA1; MYBPC1; MYBPC2; ACTN3; ACTN2; MYL1; TNNC2; TNNT3; TNNI2; NEB; MYOM2; MYH6
Lung Fibrosis	8/61	1.39E-7	1.23E-5	CEBPB; IL1B; CCL3; HMOX1; CCL2; PTX3; TIMP1; CXCL2
Myometrial Relaxation and Contraction Pathways	9/153	2.05E-5	0.00120	RYR1; MYLK2; ACTA1; IGFBP3; IL1B; RGS16; MAFF; FOS; ATF3
Oxidative Stress	4/28	1.52E-4	0.00670	HMOX1; MT1; FOS; JUNB
Spinal Cord Injury	6/99	4.32E-4	0.0152	IL1B; CCL2; CXCL1; FOS; FCGR2B; CXCL2
p53 Signaling	5/67	5.07E-4	0.0149	CDKN1A; IGFBP3; SERPINE1; THBS1; GADD45G
Oxidative Damage	4/42	7.48E-4	0.0188	C1QB; CDKN1A; TNFRSF1B; C1QC
Regulation of Cardiac Hypertrophy by miR-208	2/6	0.00140	0.0307	GJA5; HOPX
TGF Beta Signaling Pathway	4/52	0.00168	0.0328	SERPINE1; LIF; FOS; THBS1
Adipogenesis Genes	6/134	0.00209	0.0367	SOCS3; CEBPB; CEBPD; SERPINE1; LIF; OSM

Table S2. Pathway Analysis Results of Tolvaptan Treatment. The 10 most significant pathways obtained from WikiPathways analysis of differentially expressed genes after Tolvaptan treatment.

Term	Overlap	P-Value	Adjusted P-Value	Genes
Striated Muscle Contraction	16/45	6.95E-22	1.22E-19	MYOM1; MYBPC1; MYBPC2; ACTN3; ACTN2; TPM2; TNNC2; NEB; ACTA1; DES; JSRP1; MYL1; TNNT3; TNNI2; MYH6; MYH7
Cytokines and Inflammatory Response	5/27	4.28E-6	3.76E-4	IL11; CSF3; IL1B; CXCL1; CXCL3
Lung Fibrosis	6/61	2.04E-5	0.00120	CSF3; CEBPB; IL1B; CCL3; CCL2; CXCL2
ACE Inhibitor Pathway	3/9	6.18E-5	0.00272	NOS3; BDKRB2; KNG1
Spinal Cord Injury	6/99	3.08E-4	0.0109	IL1B; CCL2; CXCL1; LTB; FCGR2B; CXCL2
IL-1 Signaling Pathway	4/37	3.61E-4	0.0106	NFKBIA; IL1RN; IL1R2; IL1B
Myometrial Relaxation and Contraction Pathways	7/153	5.46E-4	0.0137	RYR1; MYLK2; ACTA1; RGS1; NOS3; IL1B; MAFF
Regulation of Cardiac Hypertrophy by miR-208	2/6	0.00123	0.0271	GJA5; MYH7
Adipogenesis Genes	6/134	0.00152	0.0297	SOCS3; CEBPB; CEBPD; SERPINE1; LIF; OSM
Chemokine Signaling Pathway	7/190	0.00192	0.0339	NFKBIA; FGR; CXCR2; CCL3; CCL2; CXCL1; CXCL2
